# Bi-level graph learning unveils prognosis-relevant tumor microenvironment patterns in breast multiplexed digital pathology

**DOI:** 10.1016/j.patter.2025.101178

**Published:** 2025-02-11

**Authors:** Zhenzhen Wang, Cesar A. Santa-Maria, Aleksander S. Popel, Jeremias Sulam

**Affiliations:** 1 Department of Biomedical Engineering, Johns Hopkins University, Baltimore, MD 21218, USA; 2 Mathematical Institute for Data Science, Johns Hopkins University, Baltimore, MD 21218, USA; 3 Department of Oncology, Johns Hopkins University, Baltimore, MD 21205, USA; 4 Sidney Kimmel Comprehensive Cancer Center, Baltimore, MD 21231, USA

**Keywords:** tumor microenvironment, prognosis, spatial analysis, graph learning, graph kernel, breast cancer, biomarker discovery, survival analysis, single-cell, interpretable AI

## Abstract

The tumor microenvironment (TME) is widely recognized for its central role in driving cancer progression and influencing prognostic outcomes. Increasing efforts have been dedicated to characterizing it, including its analysis with modern deep learning. However, identifying generalizable biomarkers has been limited by the uninterpretable nature of their predictions. We introduce a data-driven yet interpretable approach for identifying cellular patterns in the TME associated with patient prognoses. Our method relies on constructing a bi-level graph model: a cellular graph, which models the TME, and a population graph, capturing inter-patient similarities given their respective cellular graphs. We demonstrate our approach in breast cancer, showing that the identified patterns provide a risk-stratification system with new complementary information to standard clinical subtypes, and these results are validated in two independent cohorts. Our methodology could be applied to other cancer types more generally, providing insights into the spatial cellular patterns associated with patient outcomes.

## Highlights

• BiGraph systematically links local cell patterns with patient survival outcomes
• It provides data-driven and interpretable insights into cancer prognosis
• It complements and enhances risk stratification of classical clinical subtyping
• This approach is broadly applicable to other diseases and other spatial single-cell data

## The bigger picture

Breast cancer is one of the most common cancers in women and, unfortunately, telling how or when a patient will respond to a given treatment continues to be poorly understood——particularly in the cases of most aggressive breast cancer subtypes. The complex biology reflected in the tissue surrounding and including the tumor, known as the tumor microenvironment, has an important impact on the evolution of the disease and on the overall prognosis of the patient. However, this microenvironment is very complex and heterogeneous, making it difficult to extract informative quantitative measures that can be indicative of better patient outcomes. This work provides a method that learns, in an automatic manner, patterns of cell interactions in the tumor microenvironment. We show that these cell patterns characterize different groups of patients with distinct outcomes, leading to the discovery of prognostic biomarkers. This work highlights the power of data-driven methods in uncovering biologically meaningful features using breast cancer as a case study, but it is applicable to other diseases more broadly.

## Graphical abstract

**Figure:**
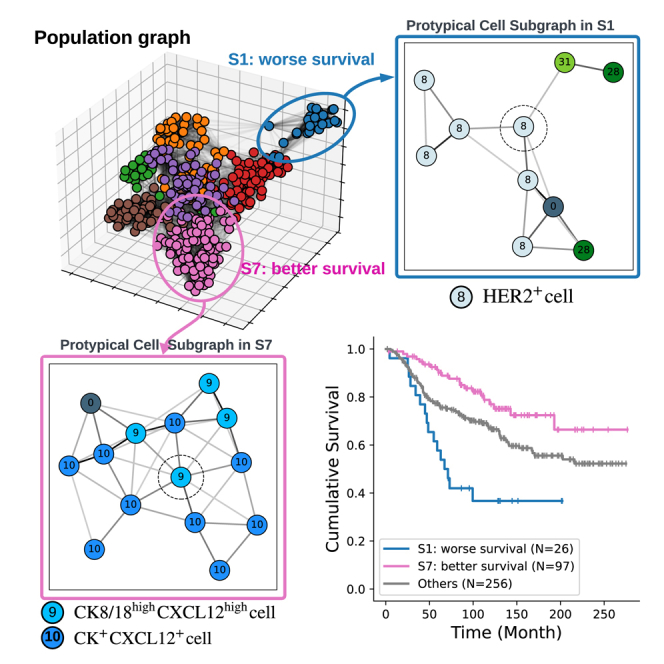

## Teaser

BiGraph provides a new approach to risk stratification and biomarker discovery in a wide range of cancers. More generally, it demonstrates the potential of data-driven methods to uncover biologically meaningful and prognostic features to study complex systems, including but not limited to tumor microenvironment.

## Introduction

The tumor microenvironment (TME) is a complex ecosystem, comprising proliferating tumor cells, tumor stroma, immune cells, blood, and lymphatic vessels.^1^ There is accumulating evidence of the pivotal role of the TME in influencing tumor progression,^2^ treatment resistance,^3^ and patient prognosis.^4^ Recent advancements in spatial multiplex proteomics technology, such as multiplexed immunohistochemistry (mIHC),^5^ co-detection by indexing (CODEX),^6^ multiplexed ion beam imaging (MIBI),^7^ and imaging mass cytometry (IMC),^8^ provide simultaneous assessment of a wide spectrum of proteins in tissue specimens. These state-of-the-art technologies provide in-depth phenotypic profiling of hundreds of thousands of cells, allowing for a comprehensive exploration of TMEs at the single-cell level.^9^

Increasing attention has been given to studying the TME at varying scales,^10^^,^^11^ including the analysis of cell types,^12^^,^^13^^,^^14^ spatial distances,^15^ and cellular neighborhoods.^16^^,^^17^^,^^18^ These works represent valuable insights across a spectrum of cancers, but associating them with clinical implications, such as treatment response and patient prognoses, remains an important and difficult challenge. Traditionally, this question relies on the formulation of explicit hypotheses based on domain expertise and prior knowledge.^12^^,^^19^^,^^20^ However, this hypothesis-driven approach naturally constrains the patterns of association that can be explored.

On the other hand, deep-learning techniques are becoming increasingly popular to study associations between the TME and disease without explicit hypotheses.^21^^,^^22^^,^^23^^,^^24^ Recent studies have reported promising results by leveraging graph neural networks (GNNs) to model the TME and predict the presence,^25^^,^^26^ grade,^27^^,^^28^ stage,^29^ subtype,^30^^,^^31^ and prognosis^32^^,^^33^^,^^34^^,^^35^^,^^36^ of different cancers. However, the limited size, biological heterogeneity, and differences in imaging protocols often result in limited generalization capacity, particularly in cross-study scenarios.^37^^,^^38^ Additionally, the interpretability of these deep and complex models remains a challenge.^39^^,^^40^^,^^41^ Many of these studies do not discuss explanations or interpretations of their models,^27^^,^^28^^,^^30^^,^^31^^,^^34^^,^^35^ some employ gradient-based^25^^,^^29^^,^^36^ and permutation-based^26^ methods to generate saliency maps, and others conduct *post hoc* analyses on learned features to obtain clinical correlates.^32^^,^^33^ These explanations, although providing some insights into the learned information, lack precise and actionable information and can be difficult to interpret to clinical experts and researchers.

**Figure 1:**
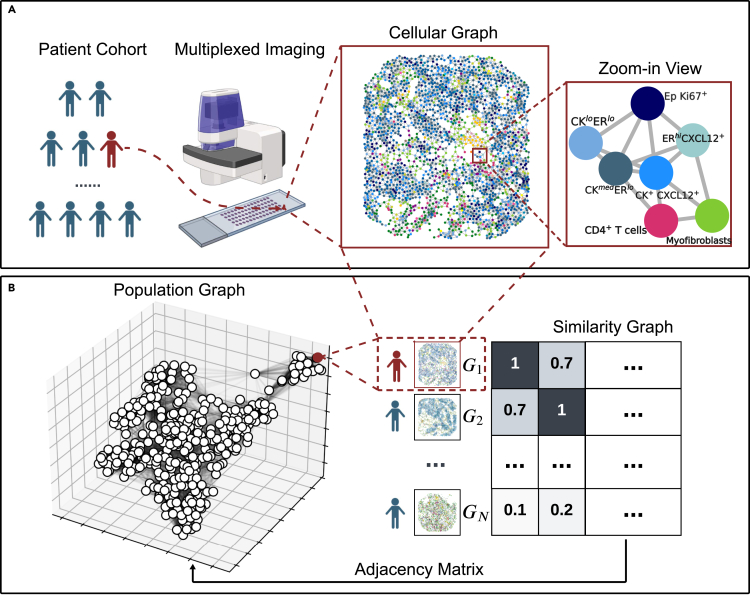
Overview of the methodology presented in this work, BiGraph (A) The cellular graph is constructed to model the tumor microenvironment (TME) of individual patients based on their multiplexed images. Each node represents a cell, and cells are connected via edges with varying weights that decrease with increasing inter-cellular distance. (B) A population graph, with patients as nodes, is constructed by measuring the inter-patient similarity based on the abundance of TME patterns.

This study presents a novel method, named BiGraph, designed to discover characteristic TME patterns associated with good and bad prognoses. BiGraph addresses two key limitations of existing methods: (1) it employs a data-driven approach instead of relying on predefined, handcrafted features typical of hypothesis-driven methods; and (2) it is fully interpretable and allows for the characterization of biomarkers, in contrast to the black-box nature of most deep-learning methods. BiGraph entails the construction of two related graphs: a patient-specific cellular graph and a population graph (see Figure 1). The former models the spatial distribution of cells in the TME for individual patients, and the latter captures similarities across all patients by comparing the prevalence of recurring cellular patterns. These recurring patterns are formally defined as “TME patterns” and may also be referred to as “cell patterns” throughout the paper.

**Figure 2:**
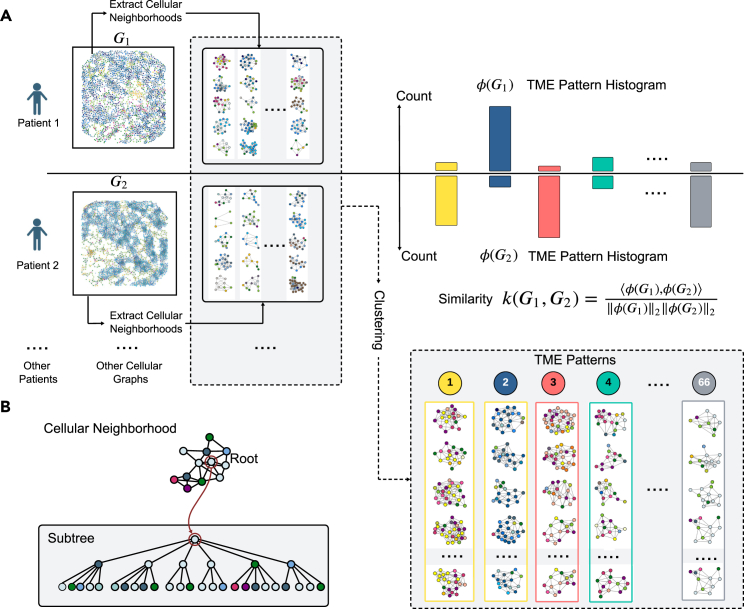
The Soft-WL subtree kernel (A) Numerous subtrees, each representing a cellular neighborhood, are extracted from cellular graphs. Similar subtrees are clustered together to form TME patterns. A histogram of TME pattern characterizes each cellular graph. The similarity between two cellular graphs, each representing a patient, is given by the cosine similarity of their pattern histograms. (B) Given a designated cell, the cellular neighborhood surrounding it forms a subtree: the designated cell is the root, and its neighbors are children nodes.

The concept of graph-in-graph learning has been applied before in designing hierarchical GNNs,^42^^,^^43^ with use cases in social networks,^42^ protein-protein interaction,^44^ and computational pathology.^30^ Unlike these, BiGraph introduces an efficient and interpretable way to connect two graphs across a hierarchy of scales through a new kernel that we dub Soft-Weisfeiler-Lehman (WL) subtree kernel. This kernel, a relaxation of the well-known WL subtree kernel,^45^ compares the abundance of cell patterns across patients (see Figure 2). This hierarchy of graphs facilitates the automatic and unsupervised risk stratification of patients by clustering them into patient subgroups with potentially different prognoses. Importantly, this risk stratification is also interpretable, as the features used to characterize each patient—the composition of their TME patterns—are transparent and biologically meaningful. We identify the most characteristic patterns for each patient subgroup, leading to explicit hypotheses on the associations between TME patterns and prognoses, which are then systematically validated in external cohorts.

While the developed methodology is general, we focus our study on breast cancer. Our analysis demonstrates that BiGraph provides a risk-stratification system for breast cancer patients that is complementary to the existing clinical subtyping system.^46^ We also report new prognostic biomarkers characterized by unique spatial cell patterns. Our findings are validated in both an inner-validation set (comprising a random subset from the discovery set) and two external-validation sets that have been independently collected and curated. These experiments showcase the generalizability of our results across diverse datasets, even with varying antigen panels and cell phenotyping systems.

## Results

In this section we present our main results, starting with a high-level overview of our methodology and a description of the datasets employed. We then demonstrate how BiGraph is applied to a breast cancer discovery cohort, reporting on the identification of TME patterns, the construction of the population graph, and the resulting risk-stratification system. We also showcase the interpretability of BiGraph by demonstrating that risk stratification can be explained by prognostic-relevant TME patterns. Finally, we thoroughly validate our findings in inner and external datasets and include comparisons with alternative approaches.

### BiGraph: Interconnected cellular and population graphs capture multi-scale information

BiGraph is designed to be applied to single-cell data obtained through multiplexed imaging technologies, and it takes the spatial coordinates and phenotypes of cells as input. As a result, it produces a risk stratification of patients together with the cell patterns that characterize each of the subgroups.

The primary innovation of BiGraph relies on exploiting relations across two graphs at different scales, a cellular graph and a population graph, by means of a graph kernel method. At the patient level, we construct a cellular graph that models the TME of each patient with each node corresponding to a cell with its phenotype label. Nodes (i.e., cells) are connected through edges representing inter-cellular interactions (see Figure 1A). Unlike conventional approaches that employ fixed distance thresholds to determine cell connectivity,^15^^,^^16^^,^^32^ we construct a complete cellular graph with the strength of interaction decreasing as the distance between cells increases (see Methods).

At the population level, inspired by the PhenoGraph algorithm,^47^ BiGraph constructs a graph where each node represents a patient, and edges between patients are determined by the similarities of their cellular graphs (see Figure 1B). Notions of similarities between (cellular) graphs can be formalized by graph kernels. In our case, this similarity is measured by a novel relaxation of the popular Weisfeiler-Lehman subtree kernel^45^ that we call Soft-WL subtree kernel. Such a kernel generates numerous subtrees from each cellular graph (each rooted at a single cell) from which “feature embeddings” are calculated by an iterative graph convolution process. These embeddings encode the cellular composition and organization within a neighborhood around the subtree root. Subtrees with similar embeddings are clustered together, representing recurring TME patterns. These patterns allow us to compute similarities between patients by comparing their prevalence in each subject’s TME (see Methods, specifically parts concerning Soft-WL subtree kernel), producing the edges in the population graph. We then employ the popular Louvain method^48^ on the resulting population graph to detect communities, or subgroups, with high inter-patient similarities (see Population graph and community detection in Methods). Importantly, the entire process is unsupervised: no information about the survival of the patients is used to obtain the population graph. Survival analysis is then applied to each patient subgroup to assess their relative risks. Furthermore, to better understand the distinct survival outcomes, we identify the most characteristic TME patterns in each subgroup and investigate their prognostic power (see Identification of characteristic patterns in Methods).

### Datasets

**Figure 3:**
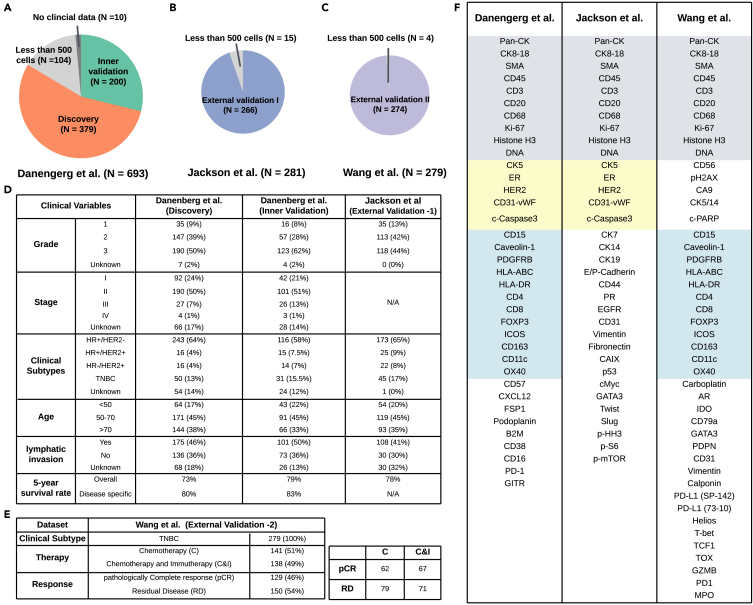
An overview of the datasets used in this work (A–C) Composition and patient splits curated by Danenberg et al.^16^ (A), by Jackson et al. (external-validation set-1)^17^ (B), and by Wang et al. (external-validation set-2)^49^ (C). (D–F) Clinical information of the discovery, inner-validation, and external-validation set-1 (D), and of external-validation set-2 (E). Antigens used in the datasets curated by Danenberg et al.,^16^ Jackson et al.,^17^ and Wang et al.^49^ Gray-shaded regions indicate the shared antigens by all three studies; yellow-shaded regions indicate the shared antigens by Danenberg et al. and Jackson et al.; blue-shaded regions indicate the shared antigens by Danenberg et al. and Wang et al. (F).

To center our study of BiGraph in the context of breast cancer, we analyze a patient cohort curated by Danenberg et al.^16^ containing 693 breast cancer patients (Figure 3B), each accompanied by their 37-dimensional IMC images of tissue microarray (TMA) cores and clinical data. This publicly available dataset also provides cell segmentation and phenotyping results (with 32 major phenotypes), as illustrated in Figure S1A. From this dataset, 10 patients with no clinical data and 104 patients with fewer than 500 cells in their TMA images are excluded. The remaining 579 patients are randomly partitioned into two subsets of 379 and 200 patients, respectively. We refer to the larger subset as the discovery set, used to develop our model, BiGraph, and to the smaller subset as the inner-validation set (see Figure 3A).

An external breast cancer patient cohort, independently curated by Jackson et al.,^17^ is used as external-validation set-1. This dataset contains data from 281 breast cancer patients (Figure 3D) with 35-dimensional IMC images of TMAs and clinical data. Cell segmentation and phenotyping results are also publicly available (see extended data in Figure S1B). Following the same patient exclusion criteria, 266 patients with more than 500 cells are included in the study (see Figure 3B). An additional external-validation cohort, independently curated by Wang et al.,^49^ is used as external-validation set-2. This dataset comprises 279 early triple-negative breast cancer (TNBC) patients who participated in a randomized pre-surgical neoadjuvant immunotherapy clinical trial (NCT02620280). Although long-term follow-up data are unavailable, information on the pathologically complete response (pCR)—defined as the absence of invasive cancer cells in post-treatment tissues—or residual disease (RD) is provided. We use this binary attribute as a surrogate for prognosis. In this cohort, tumor samples were collected at three distinct time points: before treatment, early on treatment, and post treatment. We analyze samples from the first two groups to investigate the predictive value of TME patterns in response to therapy. As before, cell segmentations and phenotyping are available (see Figure S1C), and four patients with fewer than 500 visible cells are excluded.

Notably, the two external-validation sets employ a distinct antigen panel and phenotyping system from the discovery cohort—a common challenge in current biomarker discovery research—making the validation task challenging (see Figure 3F). Fortunately, about 43% (15 out of 35) of the antigens used in the external-validation set-1 and 51% (22 out of 43) of the antigens used in the external-validation-2 are shared with that in the discovery set, as shown in the shaded regions of Figure 3F. We use the shared antigens to match the validation phenotyping systems with that in the discovery set (see Validation and generalization in Methods). For all datasets, we use the processed data provided by the sources above (i.e., cell segmentation and phenotyping results) instead of the raw IMC images as input to the BiGraph model.

### Cellular graph analysis reveals heterogeneous TME patterns

For each patient in the discovery set, a cellular graph is constructed to model the spatial distribution of different types of cells within their TMEs. The Soft-WL subtree kernel generates subtrees rooted at every single cell from each cellular graph. Each subtree is associated with a 32-dimensional feature embedding derived from an iterative graph convolution process, which encodes the composition and spatial arrangement of cells in a local cellular neighborhood surrounding the subtree root (see Soft-WL subtree kernel in Methods).

**Figure 4:**
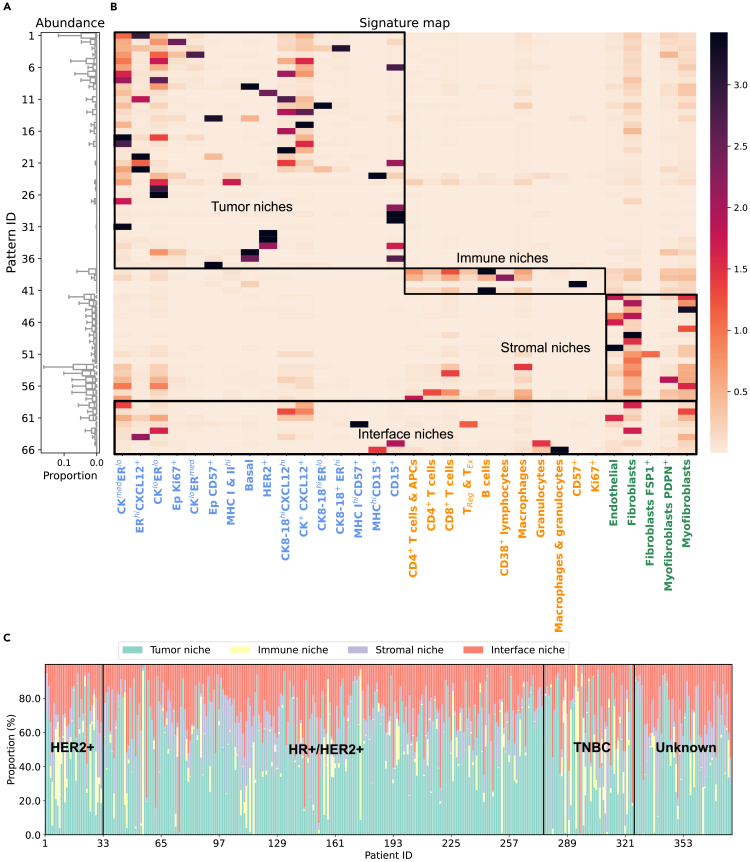
Abundance and signature map of TME patterns (A) Boxplot of the proportion of each TME pattern across patients in the discovery set. (B) Signature map of the found TME patterns; x axis, cell phenotype, with color indicating the cell type category (blue, tumor cells; orange, immune cells; green, stromal cells); y axis, pattern ID. Based on the dominating cell type, the 66 TME patterns are broadly classified into four classes: tumor niche, immune niche, stromal niche, and interface niche, as the bounding boxes indicate. (C) The proportion of tumor, immune, stromal, and interface niche patterns across all patients.

The collection of subtrees from all patients is then clustered employing their respective embeddings, resulting in 66 clusters. Each cluster is considered a TME pattern, and we refer to the centroid of each cluster as its signature, representing an abstract characterization of each pattern. Figure 4B details the signatures of the 66 found patterns and their abundance across patients in the discovery set. Based on the predominant cell phenotype, the TME patterns are grouped into four main categories: tumor niches that are mostly composed of tumor cells; immune niches dominated by immune cells; stromal niches that consist primarily of stromal cells; and interface niches, where tumor, immune, and stromal cells are mixed (Figure 4B). The vast majority of patients (i.e., *N* = 283, 75%) have all four categories present in their cellular graphs, but their relative abundance varies across patients (Figure 4C).

### Abundance of TME patterns provides inter-patient similarity

The cellular graphs described above characterize the TME of each patient with recurring patterns. To move beyond the patient level and perform a stratification analysis, we need a metric that quantifies the similarity between patients. To this end, the Soft-WL subtree kernel compares the histograms of these patterns across patients via their cosine similarity (see Methods, Equation 5). This similarity score, for any pair of patients, is in the range [0,1], with two identical graphs achieving a maximum similarity of 1.

**Figure 5:**
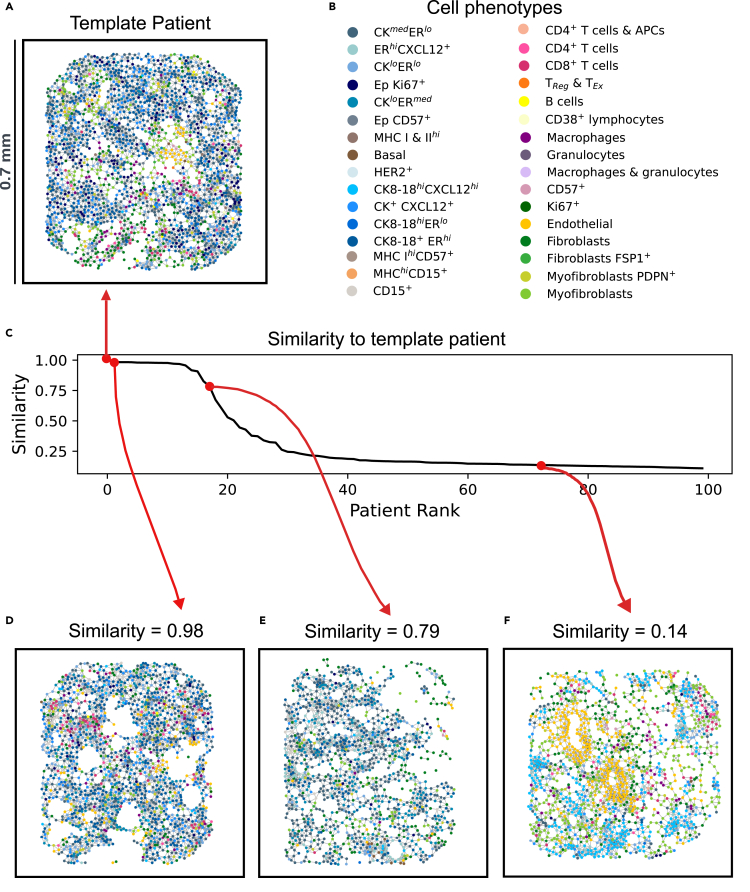
Demonstration of the inter-patient similarity as measured by the graph kernel (A) The cellular graph of a selected patient, regarded as “template” in this illustration. (B) The 32 different cell phenotypes. (C) Sorted similarity scores of other patients compared to the template patient (x axis, patient ranking; y axis, similarity score). Only the top 100 patients are shown. (D–F) Cellular graphs of patients with varying similarity scores to the template patient.

To exemplify the computation of inter-patient similarity, Figure 5A presents a randomly selected “template patient” from the discovery set for illustration purposes and depicts its cellular graph. The similarities between this patient and all others are calculated and visualized in Figure 5C (where only the top 100 are shown). Furthermore, we highlight the cellular graphs of three representative patients with varying degrees of similarity (see Figures 5D–5F). The cellular graph of the template patient contains diverse tumor cells, predominantly cytokeratin-positive cells, including $\text{CK}8/18^{+}{\text{ER}}^{\text{high}}$ cells, $\text{CK}8/18^{\text{high}}\text{CXCL}12^{\text{high}}$ cells, ${\text{CK}}^{+}\text{CXCL}12^{+}$ cells, and ${\text{CK}}^{\text{med}}{\text{ER}}^{\text{low}}$ cells. Proliferative (i.e., $\text{Ki}67^{+}$) tumor cells are also scattered throughout. Stromal cells such as fibroblasts, myofibroblasts, and endothelial cells are mixed with tumor cells, and a clear tumor-stromal boundary is difficult to discern. Immune cells, including $\text{CD}4^{+}$ T cells, $\text{CD}8^{+}$ T cells, and macrophages, are relatively rare and dispersed. One can appreciate how the patient with the highest similarity (similarity score of 0.98) exhibits nearly identical patterns to those of the template patient (see Figure 5D). Another patient with a similarity score of 0.79 (see Figure 5E) also features many interactions among various cytokeratin-positive cells, similar to those described before. However, unlike template, this patient includes many ${\text{CK}}^{\text{low}}{\text{ER}}^{\text{med}}$ cells and ${\text{ER}}^{\text{high}}\text{CXCL}12^{+}$ cells, resulting in the emergence of new patterns. Additionally, this patient exhibits a relatively immune-cold TME, with only a few immune cells infiltrating the tumor cells. In contrast, the last patient in Figure 5F has a lower similarity (0.14) and presents a vascular tumor with abundant endothelial cells. The tumor cells are relatively homotypic, dominated by $\text{CK}8/18^{\text{high}}\text{CXCL}12^{\text{high}}$ cells. This diversity in TME patterns of these four patients illustrates the capability of the Soft-WL subtree kernel to discern nuanced differences among patients’ TMEs.

### Population graph analysis provides data-driven risk stratification

Given the Soft-WL kernel as a measure of the similarity between any pair of patients, we construct a population graph that captures the inter-patient similarities across the entire set. In this graph, each node corresponds to an individual patient, and edges are assigned weights representing their similarities. A community detection algorithm is then employed to reveal patient subgroups in an unsupervised manner (i.e., without utilizing survival information; see Population graph and community detection part in Methods). The survival outcomes of these patient subgroups are then analyzed and compared with the standard clinical subtyping system. We elaborate on these results in the following subsections.

#### Patient subgroups exhibit significantly distinct survival outcomes

**Figure 6:**
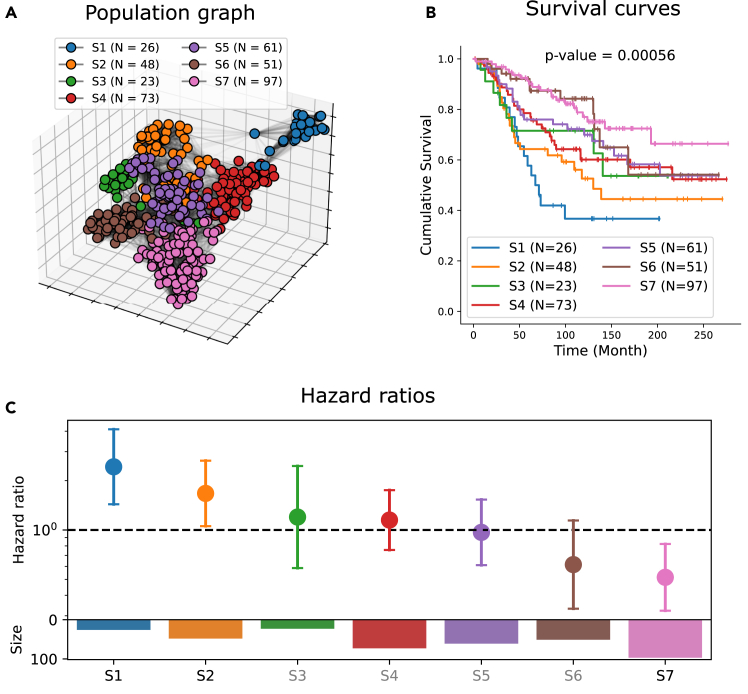
Population graph and risk stratification (A) Three-dimensional visualization of the population graph and community detection results. (B) Kaplan-Meier (K-M) survival plots for seven distinct patient subgroups. A multivariate log rank test is used to compare the survival curves. (C) Relative hazard ratios (with 95% confidence intervals) of the seven patient subgroups, estimated using a Cox proportional hazard model. The log likelihood ratio test is used to evaluate whether a hazard ratio is significantly different from one. Text color (gray vs. black) distinguishes statistically significant and non-significant associations (black, statistically significant, *p* < 0.05). The size (i.e., the number of patients) of subgroups is presented in the corresponding barplot below.

To identify patient subgroups characterized by similar TME patterns, we employ the Louvain community detection algorithm^48^ on the population graph. This process yields seven distinct communities, each representing a subgroup of patients with high intra-group similarities. These subgroups are denoted as S1–S7. The nodes in the population graph lack spatial coordinates; hence, for visualization purposes, the Fruchterman-Reingold force-directed algorithm^50^ is employed to depict a 3D representation of the entire graph. This representation and the community detection results are shown in Figure 6A.

Disease-specific survival functions of the seven patient subgroups are estimated using the Kaplan-Meier (K-M) estimator^51^ and are shown in Figure 6B. A multivariate log rank test^52^ is conducted to compare their survival outcomes, revealing a statistically significant difference (*p* = 0.00056) among the groups. Hazard ratios for these patient subgroups are calculated using the Cox proportional regression model^53^ and are presented in Figure 6C. The results reveal that S1 exhibits statistically significantly worse survival compared to other patients (hazard ratio [hr] = 2.43; 95% confidence interval [CI], 1.43–4.11; *p* = 0.0009). S2 also demonstrates significantly worse survival (hr = 1.67; 95% CI, 1.06–2.65; *p* = 0.03). Conversely, S7 exhibits statistically significantly better survival (hr = 0.51; 95% CI, 0.32–0.82; *p* = 0.005).

#### BiGraph provides complementary information to standard clinical characteristics

**Figure 7:**
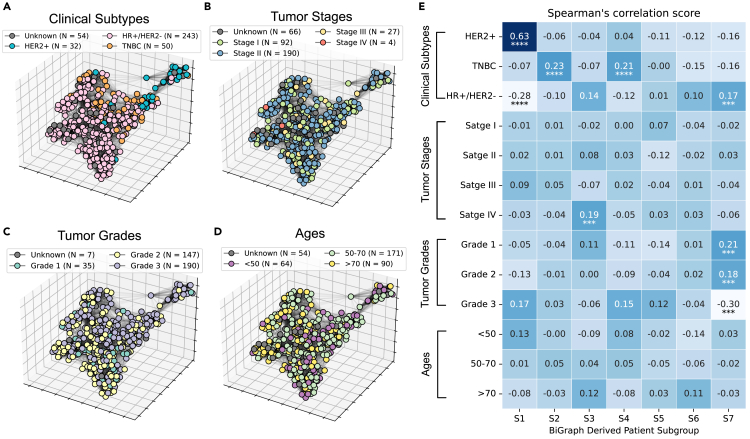
Correlation between clinical characteristics and BiGraph-derived subtypes (A–D) Population graph with nodes colored by clinical subtypes (A), by tumor stage (B), by tumor grade (C), and by age group (D). (E) Spearman’s correlation scores between clinical characteristics and BiGraph-derived subgroups. ∗∗∗∗*p* < 1e−4; ∗∗∗*p* < 1e−3.

It is natural to ask how the population graph—constructed by comparing TME patterns across patients—relates to clinical characteristics. Does it echo the information already encoded in clinical variables, or does it provide new information and insights? To answer this question, we color the nodes of the population graph with clinical subtypes (Figure 7A), tumor stage (Figure 7B), tumor grade (Figure 7C), and patients’ age (Figure 7D). These demonstrate that neighboring nodes can have different colors, implying that patients with similar TMEs do not necessarily have the same clinical attributes. Moreover, we calculate the average inter-patient similarity for each group of patients stratified based on clinical variables or BiGraph (see Table 1). The results confirm this observation, as the average inter-patient similarity within the same clinical group is only slightly higher than that of the entire heterogeneous set. HER2+ patients represent an exception, showing a higher average inter-patient similarity (0.43).

**Table 1:** Average inter-patient similarities of various clinical groups and BiGraph-derived subgroups

Clinical subtype		Stage		Grade		Age (years)		BiGraph	
HER2+	0.43	I	0.17	G1	0.25	<50	0.22	S1	0.75
TNBC	0.15	II	0.17	G2	0.20	50–70	0.28	S2	0.26
HR+/HER2−	0.20	III	0.20	G3	0.17	>70	0.16	S3	0.73
–	–	IV	0.36	–	–	–	–	S4	0.45
–	–	–	–	–	–	–	–	S5	0.32
–	–	–	–	–	–	–	–	S6	0.61
–	–	–	–	–	–	–	–	S7	0.37
Entire cohort						0.18			

We further study the alignment between BiGraph-derived subgroups and clinical subtypes by computing Spearman’s correlation scores,^54^ revealing that they are generally not aligned (see Figure 7E), with some exceptions: S1 is strongly (and positively) correlated with HER2+ patients, and negatively correlated with HR+/HER2−. Group S7, on the other hand, exhibits positive correlations with HR+/HER2−, grade 1, and grade 3 and a negative correlation with grade 3. Additionally, S2 and S4 are positively correlated with TNBC patients.

**Figure 8:**
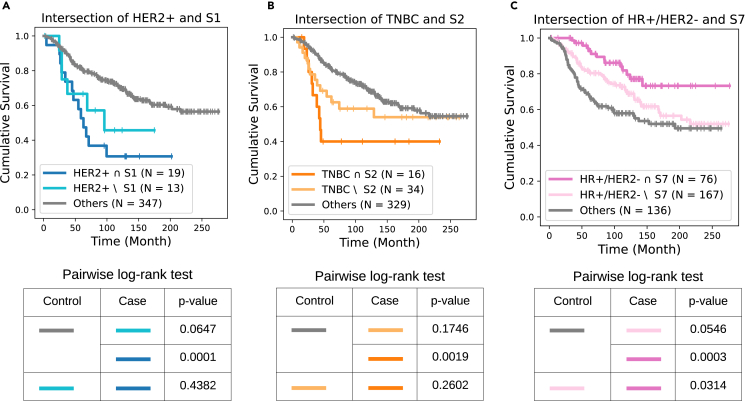
BiGraph enhances the risk stratification of clinical subtype (A) K-M survival plots of the intersection of HER2+ and S1 patients, the complement of S1 in HER2+ patients, and all other patients. (B and C) Same for TNBC and S2 (B), and for HR+/HER2− and S7 patients (C). Pairwise log rank tests are used to compare the survivals of three groups of patients in each case.

A key observation of this comparison is that the intersection between BiGraph-derived subgroups and clinical subtypes reveals unique survival patterns in some cases. For instance, HER2+ patients (*N* = 32) exhibit worse survival than others in the discovery set. Further stratification using S1 identifies a subset (*N* = 19) with even worse survival and a complement subset (*N* = 13) with slightly better survival (Figure 8A). Similarly, intersecting S2 with TNBC patients (*N* = 50) finds a subset (*N* = 16) with exceptionally worse survival outcomes (Figure 8B). Furthermore, HR+/HER2− patients (*N* = 243) display better survival outcomes, while the intersection with S7 patients (*N* = 76) shows even better survival (Figure 8C). These results suggest that the BiGraph-derived subtyping system provides complementary information to clinical subtyping, enhancing its risk-stratification capacity.

### BiGraph unveils prognosis-relevant TME patterns

The population graph illustrated above relies on comparing patients using the histogram of their TME patterns. As a result, one can directly identify patterns that are characteristic of each patient subgroup. Furthermore, the prognostic value of these patterns can be systematically investigated.

#### Patient subgroups are characterized by unique TME patterns

**Figure 9:**
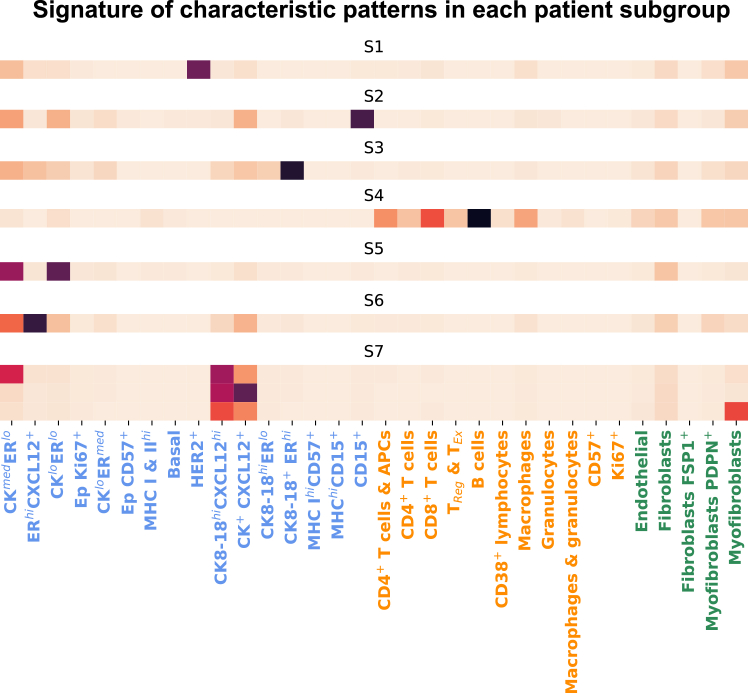
Signatures of characteristic patterns in each patient subgroup Each row presents the signature (i.e., cluster centroid) of a characteristic pattern. The x axis indicates cell phenotype, colored by category (blue, tumor cells; orange, immune cells; green, stromal cells).

**Figure 10:**
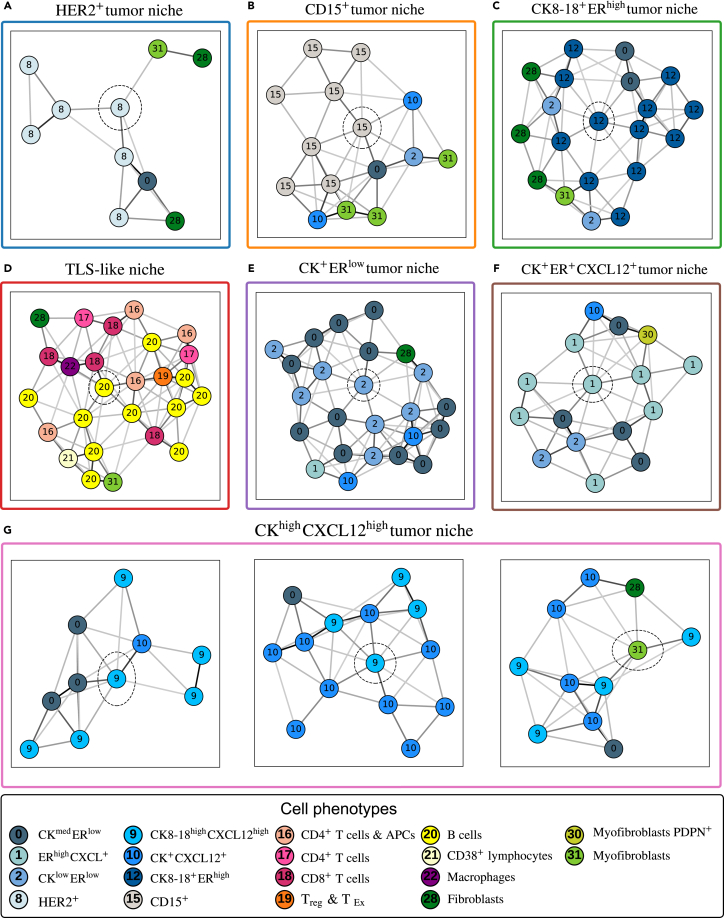
Representative examples of characteristic patterns in each patient subgroup (A) Prototypical cellular subgraphs depicting TME patterns for each patient subgroup (separated per color boxes), with cell roots circled by dotted lines. $\text{HER}2^{+}$ tumor niche, characteristic in S1. (B) $\text{CD}15^{+}$ tumor niche, characteristic in S2. (C) $\text{CK}8-18^{+}{\text{ER}}^{\text{high}}$ tumor niche, characteristic in S3. (D) TLS-like niche, characteristic in S4. (E) ${\text{CK}}^{+}{\text{ER}}^{\text{low}}$ tumor niche, characteristic in S5. (F) ${\text{CK}}^{+}{\text{ER}}^{+}\text{CXCL}12^{+}$ tumor niche, characteristic in S6. (G) ${\text{CK}}^{\text{high}}\text{CXCL}12^{\text{high}}$ tumor niche, characteristic in S7.

To identify characteristic patterns of different survival profiles, the histograms of TME patterns within and outside a patient subgroup are compared using the Hodges-Lehmann statistic.^55^ A TME pattern is deemed “characteristic” in a patient subgroup if its Hodges-Lehmann statistic surpasses 50% of the maximum value (see section Methods, and Figure S4). Results indicate that distinct patient subgroups exhibit characteristic patterns with diverse cellular compositions and organizations (see Figure 9). Representative examples of these characteristic patterns are presented in Figure 10, each centered at its root cell. Note that, while clustering is done on subtrees, we present in this figure the subgraphs from which the subtrees are extracted.

Patients in subgroup S1, exhibiting the worst survival outcome, are characterized by $\text{HER}2^{+}$ tumor niche patterns, showing enrichment of $\text{HER}2^{+}$ tumor cells surrounded by fibroblasts and myofibroblasts. The group S2, which also exhibits statistically worse survival, is characterized by $\text{CD}15^{+}$ tumor niche patterns, featuring enriched $\text{CD}15^{+}$ and other tumor cells. S3, a relatively small and homogeneous patient subgroup, is characterized by an enriched $\text{CK}8-18^{+}{\text{ER}}^{\text{high}}$ tumor niche. The most distinct pattern within S4 closely resembles the well-known tertiary lymphoid structure (TLS)^56^ and is termed TLS-like niche. This pattern encompasses a variety of immune cells, including B cells (predominantly represented), $\text{CD}4^{+}$, $\text{CD}8^{+}$, regulatory T (Treg) cells, macrophages, and $\text{CD}38^{+}$ lymphocytes.

The most characteristic pattern in S5 is a ${\text{CK}}^{+}{\text{ER}}^{\text{low}}$ tumor niche, exhibiting a mixture of ${\text{CK}}^{\text{med}}{\text{ER}}^{\text{low}}$ and ${\text{CK}}^{\text{low}}{\text{ER}}^{\text{low}}$ tumor cells. The group S6 is characterized by a ${\text{CK}}^{+}{\text{ER}}^{+}\text{CXCL}12^{+}$ tumor niche pattern, featuring the co-localization of ${\text{CK}}^{\text{med}}{\text{ER}}^{\text{low}}$ and ${\text{ER}}^{\text{high}}\text{CXCL}12^{+}$ tumor cells. Lastly, S7, the patient subgroup exhibiting the best survival outcome, presents three characteristic patterns with a common feature: a mixture of $\text{CK}8-18^{\text{high}}\text{CXCL}12^{\text{high}}$ cells and ${\text{CK}}^{+}\text{CXCL}12^{+}$ cells. Therefore, these three patterns are consolidated into one and dubbed ${\text{CK}}^{\text{high}}\text{CXCL}12^{\text{high}}$ tumor niche.

#### Prognostic value of the characteristic TME patterns

The results above showcase the most characteristic TME patterns in each subgroup, but these do not necessarily imply associations with prognosis. This naturally leads to the exploration of potential associations between these characteristic TME patterns and patient survival outcomes.

**Figure 11:**
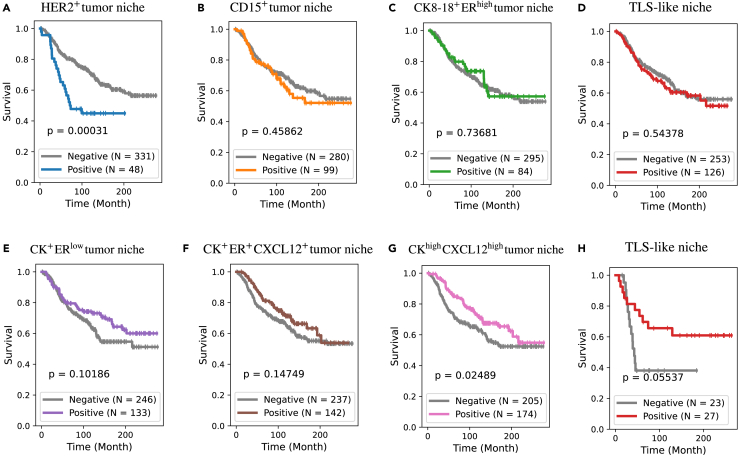
Prognostic impact of characteristic TME patterns (Patients are categorized into “positive” and “negative” groups based on the proportion of a designated TME pattern (with 1% as threshold). Survival outcomes are compared by a log rank test, and the resulting *p* values are indicated in each corresponding figure. Risk stratification of $\text{HER}2^{+}$ tumor niche (A), $\text{CD}15^{+}$ tumor niche (B), $\text{CK}8-18^{+}{\text{ER}}^{\text{high}}$ tumor niche (C), TLS-like niche (D), ${\text{CK}}^{+}{\text{ER}}^{\text{low}}$ tumor niche (E), ${\text{CK}}^{+}{\text{ER}}^{+}\text{CXCL}12^{+}$ tumor niche (F), and ${\text{CK}}^{\text{high}}\text{CXCL}12^{\text{high}}$ tumor niche (G). Risk stratification of TLS niche, specifically in TNBC patients (H).

We assess the prognostic impact of a specific TME pattern by categorizing patients as “positive” or “negative” based on the relative presence of that pattern. A 1% threshold is employed for patient stratification (the same as the cutoff point used in the definition of hormone receptor [HR] status^57^). For each characteristic pattern, survival outcomes of positive and negative patients are compared using the log rank test. Results indicate that the $\text{HER}2^{+}$ tumor niche, characteristic of S1, is associated with worse survival (*p* = 0.00031) (Figure 11A). In contrast, the ${\text{CK}}^{\text{high}}\text{CXCL}12^{\text{high}}$ tumor niche, characteristic of S7, is associated with better survival (*p* = 0.025) (Figure 11G). For patterns characteristic in S5 and S6, positive patients exhibit slightly better survival outcomes than negative patients, although the differences are not statistically significant. These results are broadly consistent with the risk stratification provided by the seven patient subgroups: S1 patients display significantly worse survival outcomes, and its most characteristic TME pattern proves to be a negative prognostic factor. Conversely, S7 patients exhibit significantly better survival outcomes, with its most characteristic TME pattern emerging as a positive prognostic factor.

Moreover, since TNBC is an especially aggressive and challenging breast cancer subtype, we further examine the prognostic values of these patterns within TNBC patients. Interestingly, the TLS-like niche, characterized by densely aggregated B cells surrounded by $\text{CD}4^{+}$ and $\text{CD}8^{+}$ T cells, macrophages, and stromal cells, shows no prognostic value across the entire discovery set (*N* = 379) with heterogeneous clinical subtypes (Figure 11D). However, in the TNBC subset (*N* = 50), patients with TLS-like niches present in their TMEs do exhibit better survival than negative patients (Figure 11H). Although this difference is not statistically significant, likely due to the small cohort size, the large divergence of the two survival curves is noteworthy.

**Figure 12:**
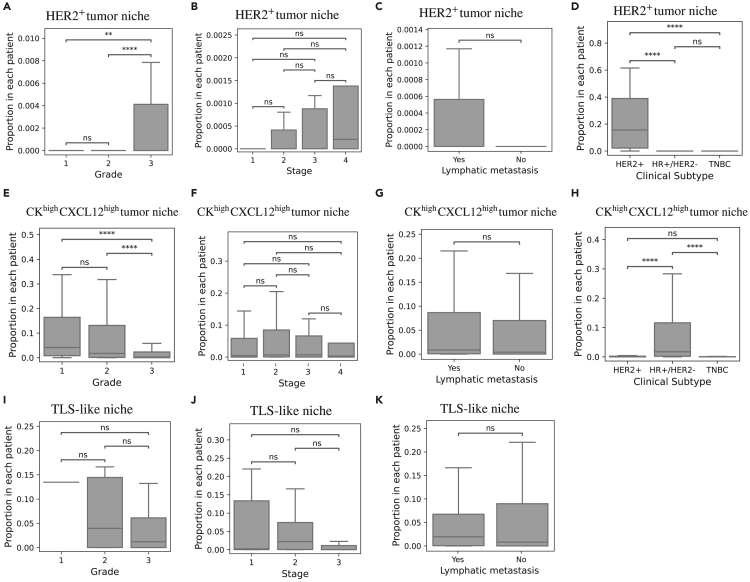
Comparison between prognosis-relevant TME patterns and clinical variables (A–D) Boxplots of the proportion of $\text{HER}2^{+}$ tumor niche in different tumor grades (A), tumor stages (B), metastasis status (C), and clinical subtypes (D). (E–H) Boxplots of the proportion of ${\text{CK}}^{\text{high}}\text{CXCL}12^{\text{high}}$ tumor niche in different tumor grades (E), tumor stages (F), metastasis status (G), and clinical subtypes (H). (I–K) Boxplots of the proportion of TLS-like niches in different tumor grades (I), tumor stages (J), and metastasis status (K). Only TNBC patients are included in TLS-like niches. Outliers are not shown in the boxplots. Mann-Whitney U test is used to compare proportions. ∗∗∗∗*p* < 1e−4; ∗∗∗*p* < 1e−3; ∗∗*p* < 1e−2.

To better understand the prognostic value of TME patterns, we investigate their correlation with standard clinical variables, such as tumor grade, stage, metastasis, and clinical subtypes. Results suggest that the expression of $\text{HER}2^{+}$ tumor niche is significantly higher in grade 3 patients (see Figure 12A) and in Her2+ patients (see Figure 12D). In contrast, the expression of ${\text{CK}}^{\text{high}}\text{CXCL}12^{\text{high}}$ tumor niche is significantly lower in grade 3 patients (see Figure 12E) but significantly higher in HR+/HER2− patients (see Figure 12F). These findings highlight the association between HER^+^ tumor niches with more aggressive clinical features (i.e., higher grade and HER2+ subtype) and the association between ${\text{CK}}^{\text{high}}\text{CXCL}12^{\text{high}}$ tumor niches and less aggressive features (i.e., lower grade and HR+/HER2− subtype). In the case of TLS-like niches, we limit the analysis to TNBC patients (given they are associated with better survival only in this subset). Results show no significant correlation between TLS-like niches and grade, stage, or metastasis (see Figures 12J and S12E).

### Validation and generalization

As detailed in the previous section concerning datasets, a subset comprising 200 patients is reserved from the 579 patients curated by Danenberg et al.,^16^ denoted as the inner-validation set. Additionally, an external breast cancer patient cohort curated by Jackson et al.,^17^ referred to as the external-validation set-1, and an additional external TNBC patient cohort curated by Wang et al.,^49^ referred to as the external-validation set-2, are utilized to evaluate BiGraph’s cross-study generalization capacity. Importantly, these validation sets allow us to explore generalization under different settings. The inner-validation set provides generalization results to data collected under the same protocol as those employed in the discovery set, whereas the other two external-validation sets involve data collected independently, employing a distinct antigen panel and phenotyping system.

To address this challenge, we align the cell phenotyping in the external-validation set with that of the discovery set. This alignment is achieved by identifying shared antigens between both sets, as indicated by shaded regions in Figure 3F. More precisely, every cell in the external-validation set is mapped to the closest cell phenotype cluster in the discovery set, where the closeness is measured by the Euclidean distance of their expressions of the shared antigens (see Validation and generalization in Methods). The quality of the resulting cell phenotyping alignment is shown in Figure S5). With the cell phenotyping systems aligned, we validate the major results from the previous sections in both inner- and external-validation sets.

#### Validation of risk stratification

**Figure 13:**
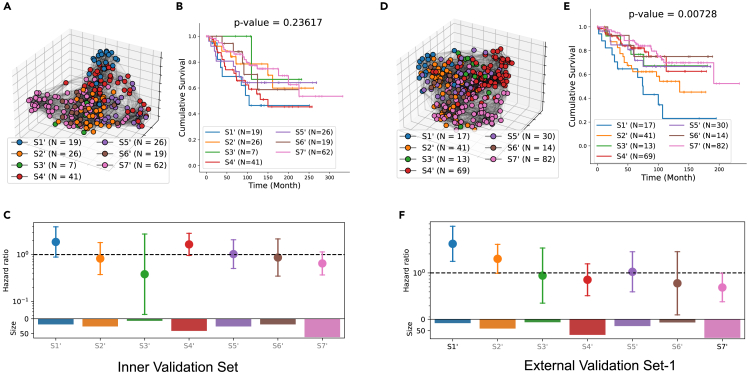
Validation of the risk-stratification results (A–C) Results in inner-validation set (*N* = 200). Population graph with nodes colored by their assigned patient subgroup (A). K-M survival functions of seven mapped patient subgroups. A multivariate log rank test is used to compare them, and the *p* value is indicated in the title (B). Hazard ratios (with 95% confidence interval) of the seven mapped patient subgroups, with color distinguishing between statistically significant (black, *p* < 0.05) and non-significant (gray) associations. The log likelihood ratio test is used to evaluate whether a hazard ratio is significantly different from one. The size of each subgroup is presented in the corresponding barplot below (C). (D–F) Results in external-validation set-1 (*N* = 266), with the same layout as (A)–(C).

Our results in previous sections identified seven patient subgroups from the population graph of the discovery set with significantly distinct survivals. In the validation stage, every new patient (with their corresponding cellular graph) is mapped to one of the seven patient subgroups using a k-nearest neighbor (k-NN) rule.^58^ This assignment requires a notion of distance, for which we use the Soft-WL subtree kernel, defined previously. In this way, we identify seven patient subgroups in each inner and external-validation sets, denoted by $S1^{\prime },S2^{\prime },\ldots$, S $7^{\prime }$. Note that the driving characteristics of the seven subgroups are solely determined by the data in the discovery set. We analyze the survival outcomes of the seven mapped patient subgroups in both inner (Figures 13A–13C) and external-validation set-1 (Figures 13D–13F). The validation of risk stratification on the external-validation set-2 (from a clinical trial) is not feasible, since long-term survival data are unavailable. A multivariate log rank test is used to compare the K-M survival plots of the seven patient subgroups.

The survival outcomes of the seven mapped patient subgroups do not have a statistically significant difference (*p* = 0.24, Figure 13B) in the inner-validation set, but they do in the external set (*p* = 0.007, Figure 13E). By comparing the hazard ratios of the seven mapped (validation) subgroups with those in the discovery set (Figure 6C), we observe a consistent trend from $S1^{\prime }$ to $S7^{\prime }$, supporting the reproducibility of our findings. Furthermore, our validation results confirm that $S1^{\prime }$ in the external-validation set exhibits significantly worse survival (*p* ⟨ 0.05), consistent with the prognosis of S1 in the discovery set. Similarly, $S7^{\prime }$ in the external-validation set demonstrates better survival outcomes (*p* ⟨ 0.05), mirroring the survival pattern of S7 observed in the discovery set. In the inner-validation set, $S1^{\prime }$ and $S7^{\prime }$ have a relatively high and low hazard ratio, respectively, while both lack statistical significance.

**Figure 14:**
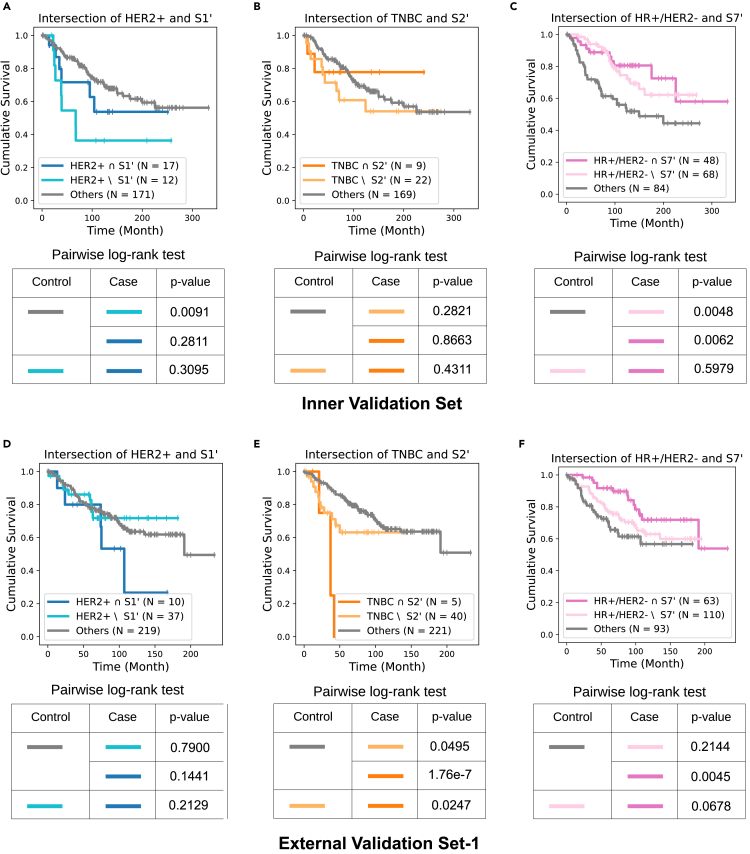
BiGraph of enhanced risk stratification when combined with clinical subtyping (A) BiGraph provides enhanced risk stratification when combined with clinical subtyping: validation results. (B and C) Survival plots of the intersection of HER2+ and $S1^{\prime }$ patients, the complement of $S1^{\prime }$ in HER2+ patients, and all other patients. Analogous survival for TNBC and $S2^{\prime }$ (B), and HR+/HER2− and $S7^{\prime }$ patients (C). (D–F) Pairwise log rank tests are used to compare the survivals of three groups of patients. Results of external-validation set, with the same layout as (A)–(C).

Recall that we found the intersection between a clinical subtype and a BiGraph-derived subgroup exhibits exceptionally better (or worse) survivals in some cases in the discovery set (Figure 7). We now validate this observation in the validation sets. While the intersection of HER2+ and $S1^{\prime }$ patients (Figures 14A and 14B), and that of TNBC and $S2^{\prime }$ patients (Figures 14B and 14E), show contradictory results, the intersection between HR+/HER2− and $S7^{\prime }$ patients (Figures 14C and 14F) indeed has exceptionally better survival than the complement of S7 in the HR+/HER2− group and patients with other clinical subtypes. This is not statistically significant, however, likely due to differences in data acquisition protocols (e.g., different antigen panels), potential distribution shifts, and the smaller size of the validation sets, all of which represent challenges to validating the findings from the discovery set.

#### Validation of the prognostic values of TME patterns

As detailed in the previous section on the prognostic values of TME patterns, we find two prognostic-relevant TME patterns in the discovery set: the $\text{HER}2^{+}$ and the ${\text{CK}}^{\text{high}}\text{CXCL}12^{\text{high}}$ tumor niches, associated with worse and better survival, respectively. We will now validate these findings in both the inner-validation and external-validation set-1. Moreover, recall that, while the TLS-like niche is not associated with a distinct prognosis across the entire discovery set, it is linked to better survival in TNBC patients. Therefore, this pattern is validated in the TNBC subset of validation sets.

To estimate the expression of these patterns in the validation sets, we map the subtrees derived from their cellular graphs to the closest TME pattern. Then, the signatures of each TME pattern in the discovery and validation sets are qualitatively compared to assess the TME pattern mapping quality (see Figure S6). Furthermore, the proportions of the TME pattern across discovery and validation sets are compared to identify any potential distribution shifts (see Figure S7). Patients in the validation sets are categorized as positive or negative based on the expression level of the studied TME pattern, with a 1% proportion as the cutoff, as before. Notably, while the discovery set and external-validation set-1 consist of observational series where patients received standard care, external-validation-2 is a clinical trial where patients received chemotherapy with or without immune therapy (C vs. chemotherapy and immunotherapy [C&I]). Since long-term follow-up data are unavailable for this set, pathological response post-treatment serves as a surrogate for prognosis. In this case, we compare the proportion of TLS-like niches for patients who achieved pCR versus those with RD.

**Figure 15:**
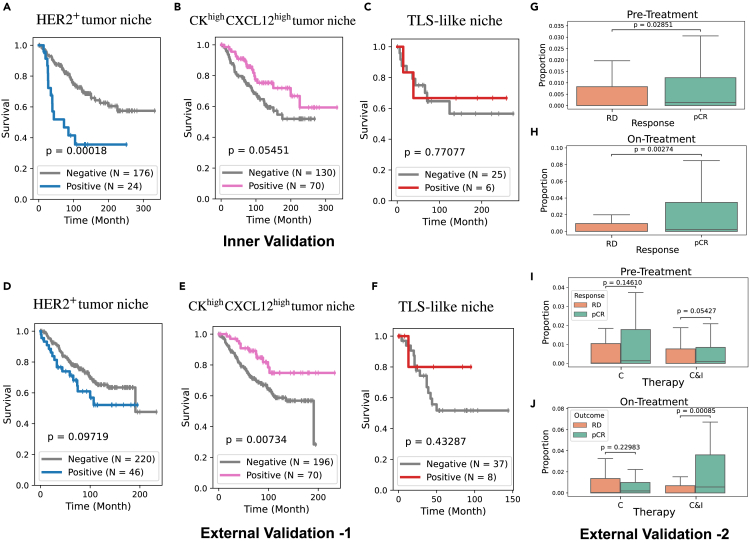
Validation of prognosis value of TME patterns (A–C) K-M survival plots of patients with (proportion ≥ 1%) and without (respectively, < 1%) $\text{HER}2^{+}$ tumor niche patterns (A) and ${\text{CK}}^{\text{high}}\text{CXCL}12^{\text{high}}$ tumor patterns (B) across patients in the validation set. Analogous to those with and without TLS-like niche patterns in TNBC patients in the inner-validation set (C). A pairwise log rank test is used to compare them. (D–F) Same analysis as (A)–(C) but on external-validation-1. (G–H) Proportion of TLS-like niche patterns in pre-treatment (G) and on-treatment (H) patients with residual disease (RD) and pathologically complete response (pCR) in external-validation set-2. (I–J) Boxplots of the proportions of TLS-like niche patterns in pre-treatment (I) and on-treatment (J) samples for patients with RD or pCR in external-validation set-2, stratified by treatment type: chemotherapy (C) vs. chemotherapy and immunotherapy (C&I). Mann-Whitney U test is used to test for differences in samples. Outliers are not shown.

Results show that patients positive for $\text{HER}2^{+}$ tumor niche indeed exhibit worse survival outcomes than those in the negative group, in both the inner-validation set (*p* = 0.0002) and external-validation set (*p* = 0.097), with only the former being statistically significant (Figures 15A and 15D). On the other hand, ${\text{CK}}^{\text{high}}\text{CXCL}12^{\text{high}}$ tumor-niche-positive patients demonstrate better survival outcomes in both inner (*p* = 0.055) and external (*p* = 0.007) validation sets (Figures 15B and 15E).

For the TLS-like niche in TNBC patients, the inner-validation set and external-validation set-1 show a slight trend toward better survivals for TLS-like-positive patients, though the differences are not statistically significant—likely due to the small size of 31 and 45 TNBC patients in these sets (Figures 15C and 15F). In the external-validation set-2, the proportion of TLS-like niche is assessed in the tissue samples collected before (i.e., pre-treatment samples) and at the early stage of the treatment (i.e., on-treatment samples). The results indicate that, in both pre-treatment and on-treatment samples, the proportion of TLS-like niche is statistically significantly higher in patients with pCR (better outcome) compared to those with RD (worse outcome) (Figures 15G and 15H). Specifically, a higher presence of TLS-like niches in on-treatment samples strongly indicates better outcomes for patients receiving C&I (Figure 15J).

**Figure 16:**
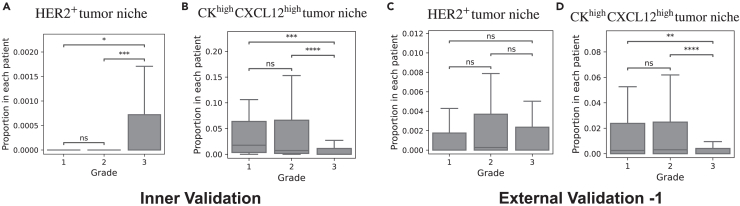
Analysis of TME patterns and tumor grade in validation sets Boxplots of the proportion of $\text{HER}2^{+}$ tumor niche pattern per patient for tumor grades 1, 2, and 3 in the inner-validation set (A) and external-validation set-1 (C), respectively. Boxplots of the proportion of ${\text{CK}}^{\text{high}}\text{CXCL}12^{\text{high}}$ tumor niche patterns per patient for tumor grades 1, 2, and 3 in inner-validation set in inner (B) and external-validation set-1 (D), respectively. Outliers are not shown. Mann-Whitney U test is used to compare proportions. ∗∗∗∗*p* < 1e−4; ∗∗∗*p* < 1e−3; ∗∗*p* < 1e−2.

Before moving on, we also validate the association between the patterns above and tumor grade. The $\text{HER}2^{+}$ tumor niche is statistically over-expressed in grade 3 patients in the inner-validation set (Figure 16A), consistent with the results on the discovery set. However, there are no significant differences in the external-validation set (Figure 16C). On the other hand, the results in both inner and external-validation sets confirm that the ${\text{CK}}^{\text{high}}\text{CXCL}12^{\text{high}}$ tumor niche pattern is statistically under-expressed in grade 3 patients (Figures 15B and 15D), supporting our findings.

### Comparison with alternative methods

The primary insight behind BiGraph is to establish a link between patient-specific cellular features (based on the spatial distribution of cell types) and the population-level groups of patients by utilizing a graph kernel function. As explained above, this is done by the Soft-WL subtree kernel, which measures inter-patient similarity by assessing the distribution of TME patterns within different TMEs. This naturally prompts the question: are there alternative approaches to measuring inter-patient similarity that could lead to conclusions similar to the ones presented in the previous section? To answer this question, we keep the construction of a population graph, community detection, and survival analysis consistent with the previous experiments but explore alternative methods to calculate inter-patient similarities. We consider the following alternatives and comment on the corresponding results for each case.

**Figure 17:**
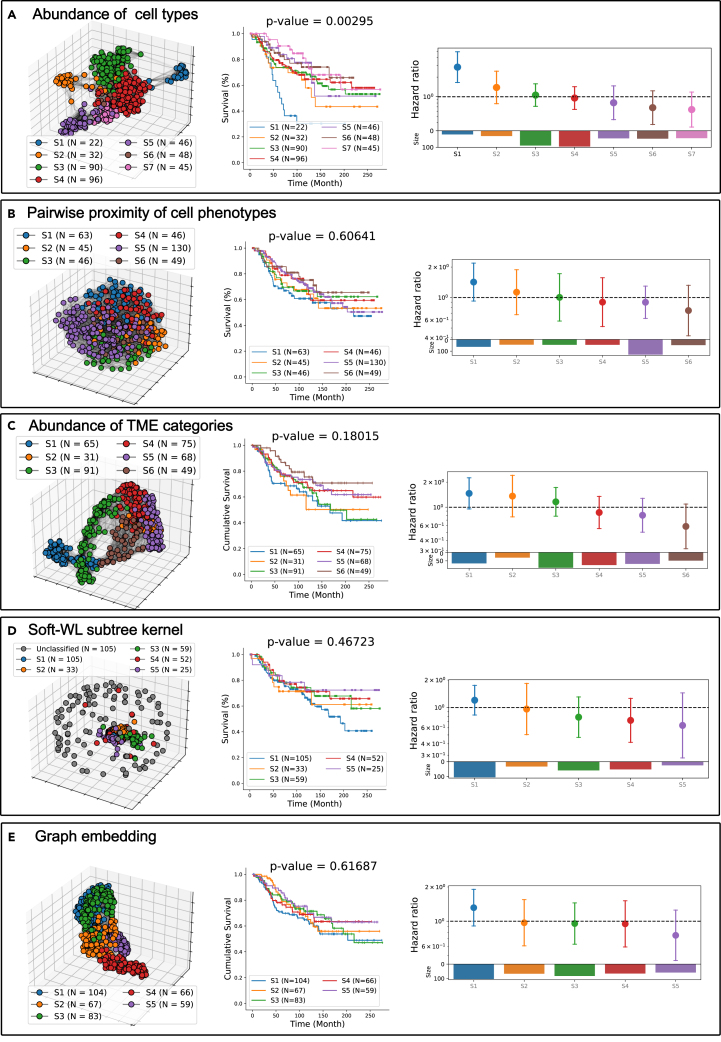
Clustering and risk stratification of comparative methods on the discovery set Risk-stratification results from characterizing patients by the abundance of cell types on the discovery set (A). First column, population graph with nodes colored by patient subgroup labels; second column, K-M survival plots of patient subgroups, a multivariate log rank test is used to compare them, and the *p* value is indicated in the title; third column, hazard ratios (with 95% confidence interval) estimated by the Cox proportional model. The log likelihood ratio test is used to evaluate whether a hazard ratio is significantly different from one. Analogous results by characterizing patients through pairwise distance of cell types (B), abundance of four TME categories (C), conventional WL subtree kernel (D), and a graph embedding method (E), respectively.

Abundance of cell types: In this approach, each patient is simply characterized by their cell composition, given by a histogram, and the inter-patient similarity is determined by the cosine similarity of the corresponding histograms. Thus, this approach disregards the spatial information and interactions between cells. The community detection algorithm identifies seven patient subgroups in the population graph, denoted by S1 to S7. Notably, only S1 exhibits significantly worse survival (see Figure 17A). The S1 subgroup identified here and the one found by the Soft-WL subtree kernel reveal a large overlap, with an intersection over union (IoU) score of 0.85 – as with the previous result, the former is also characterized by an over-presentation of $\text{HER}2^{+}$ cells. On the other hand, the subgroup S7 identified by the Soft-WL subtree kernel as having better survival and characterized by TME patterns involving multiple types of cells does not align with any of the subgroups identified using the abundance of cell types.

Pairwise proximity of cell types: **i**n this approach, each patient is characterized by the average proximity score between any possible pairs of cells of different phenotypes (see Equation 6). This proximity score increases as the spatial distance between cells decreases. While this method considers spatial information to some extent, it is limited to pairwise relationships. As can be seen in Figure 17B, the resulting population graph does not demonstrate any clear clusters, and the community detection algorithm identifies six patient subgroups, none of them exhibiting any distinct survival outcomes.

Abundance of TME categories: recall from the cellular graph analysis that the 66 TME patterns are grouped into four main categories (tumor niches, immune niches, stromal niches, and interface niches) based on the predominant cell phenotypes within each subtree. For this comparison, we describe each patient by a histogram of these four TME categories, and the inter-patient similarity is determined by the cosine similarity of their histograms. This approach is a similar but coarser version of BiGraph since fine-grained differences among TME patterns are omitted. The resulting population graph unveils six patient subgroups, but none of these exhibit distinct survival outcomes (see Figure 17C).

WL subtree kernel: **t**he Soft-WL subtree kernel, presented in this work, measures inter-patient similarity by identifying identical TME patterns across patients, each corresponding to a cluster of similar subtrees. In contrast, the classic WL subtree kernel^45^ iteratively identifies isomorphic subtrees across graphs (see Comparative methods in Methods). We explore two formulations of the WL subtree kernel: one that considers isomorphic subtrees found in all previous iterations and accumulates these similarities (see Equation 8) and another that only considers isomorphic subtrees found at the last iteration (see Equation 9). Results indicate that the former version yields almost the same results as characterizing patients by the abundance of cell types (see Figure S8), likely because of the stringent criteria of the subtrees being isomorphic. As iterations progress, the depth of subtrees to consider increases, and isomorphic subtrees become rare. Thus, iteration zero (where a subtree is a single node) dominates the similarity score. This phenomenon also poses challenges to the latter formulation by leading to the diagonal dominance problem,^59^ where a cellular graph is only identical to itself. The corresponding population graph shows many isolated nodes, representing patients dissimilar to all others. The community detection algorithm identifies five patient subgroups, none with distinct survival outcomes (see Figure 17D).

These results suggest that the stringent comparison employed in the conventional WL subtree kernel yield similarity metrics that prevent us from discovering any relevant patient subgroups. Additionally, the feature space of the WL subtree kernel has an extremely high dimension (see Figure S9), making the interpretation (i.e., identification of characteristic subtrees) prohibitive.

Graph embedding: **l**astly, we use the FEATHER^60^ graph embedding algorithm to generate a low-dimensional vector for each cellular graph (i.e., for each patient). The inter-patient similarity is then determined by the cosine similarity of the resulting vectors. FEATHER models the spatial information and inter-cellular interactions using characteristic functions of the node features. As a result, the resulting embeddings are not interpretable, and thus no explicit characteristic patterns can be identified. For this case, the population graph (Figure 17E) reveals clearer clusters than those arising from the pairwise proximity of cell phenotypes (Figure 17B) and the WL subtree kernel (Figure 17D). However, while the community detection algorithm identifies five patient subgroups, none of these show distinct survival outcomes.

## Discussion

The TME represents a fundamental piece in the study of cancer biology, and several recent studies indicate its promise for discovering biomarkers for prognosis and even treatment response.^3^^,^^4^^,^^19^^,^^20^^,^^61^^,^^62^^,^^63^ However, the number of generalizable biomarkers from pathology that provide strong prognosis information remains limited. Additionally, while some useful biomarkers have been derived from pathology samples, such as the tumor-infiltrating lymphocytes (TILs) score,^64^^,^^65^ the tumor-immune mixing score,^15^ the presence of TLS,^56^^,^^66^ among others, they rely heavily on traditional segmentation tools,^67^ handcrafted spatial statistics,^15^ and hypotheses limited to those arising from domain expertise. The discovery of data-driven biomarkers for prognosis has remained unexplored. Modern deep-learning methods, including GNNs, constitute an appealing alternative for data-driven biomarkers for prognosis.^21^^,^^22^ However, their use in real-world clinical settings remains constrained by the lack of rigorous external validation^37^^,^^38^ and limited explainability.^27^^,^^28^^,^^30^^,^^31^^,^^34^^,^^35^

This study introduces a data-driven methodology centered on breast cancer that relies on two simple observations: (1) representative phenotypic patterns of the TME can be adaptively learned by analyzing patient-specific cellular graphs constructed from multiplexed tissue image data, and (2) the relative abundance of such patterns in different patients can be employed to provide a measure of their similarity, from which a population-level graph can be constructed. This bi-level process allows us to obtain—automatically and in an unsupervised manner—different patient subgroups with different prognoses. Compared with other data-driven methods, such as the ones mentioned above, our approach provides complete transparency of the features that provide risk stratification. These features, which we dub TME patterns, represent similar subtrees in the TME, each corresponding to a local cellular neighborhood with unique cellular composition and spatial organization. As a result, we can easily analyze cell patterns that are over-expressed in groups with better (and worse) survival. Relying on more complex models, such as those based on GNNs, would have made it significantly more challenging—if not altogether impossible—to characterize such biomarkers clearly and easily.

Another contribution of this work is the formalization of the Soft-WL subtree kernel, a novel relaxation of the WL subtree kernel.^45^ This kernel method efficiently captures and compares local structures in complex cellular graphs with numerous cells and various phenotypes. As shown in the comparison with alternative methods, it over-performs other simpler methods, such as simply quantifying the abundance of different types of cells, by considering their spatial co-localization and interactions. On the other hand, it alleviates the diagonal dominance issue of the conventional WL subtree kernel,^59^ providing a smoother comparison of subtrees. On the other hand, a limitation of our method is the over-smoothing phenomenon,^68^^,^^69^ which is shared by many other graph learning methods. As the graph convolution progresses and subtree depth increases, subtrees tend to become increasingly similar to each other, preventing us from capturing higher-order structures in the graphs. We believe that BiGraph could be improved further, for example by replacing the Soft-WL subtree kernel with more advanced graph kernel methods.

This study provided a data-driven risk stratification for cohorts of breast cancer patients, featuring seven subgroups with varying prognostic outcomes. This risk-stratification scheme is furthermore shown to be robust in both an inner-validation set (a subset of patients randomly selected from the original cohort) and two external-validation sets (independent datasets collected with different acquisition protocols and antigen panels). Patients in all validation sets mirror the prognosis of those with similar TMEs in the discovery set. Furthermore, and importantly, by comparing this new risk-stratification system with the standard clinical subtyping system based on HR and HER2, we found that ours provides complementary information that enhances the risk-stratification capacity of conventional guidelines.

By analyzing the TME patterns that characterize each patient subgroup, we find that $\text{HER}2^{+}$ tumor niches are strongly associated with worse survival, higher tumor grade, and the clinically defined HER2+ subtype. These findings are validated in independent, external-validation cohorts. As shown in many previous works, the over-expression of HER2 has been associated with a more aggressive breast cancer phenotype and with decreased survival.^70^^,^^71^^,^^72^^,^^73^ Our study is consistent with this observation, but this is found in an entirely data-driven manner. On the other hand, we found that tumor niches expressing high levels of cytokeratins and CXCL12 are associated with better outcomes, lower tumor grade, and the clinically defined HR+/HER2− subtype. This finding is particularly noteworthy as the prognostic impact of the interplay between these proteins, especially in breast cancer, is underexplored. While some literature discusses their individual implications, the evidence on their prognostic impact is still controversial: some demonstrate its association with the downregulation of genes linked to metastasis inhibition, promoting the invasion and migration of breast cancer cells,^74^^,^^75^ while others suggest that higher expression of CXCL12 is associated with better survival, suppressing tumor growth and invasion in both breast^76^ and pancreatic cancer.^77^ The prognostic impact of cytokeratin is even more complicated due to the diverse range of subtypes. CK5/6, CK14, and CK20 are known to be positively associated with high tumor grade of breast cancer,^78^^,^^79^ and CK17 is also linked to poor clinical outcomes in breast cancer patients.^80^ Conversely, downregulation of CK8/18 has been reported to promote breast cancer progression, implying a favorable prognostic impact of CK8/18.^81^^,^^82^^,^^83^ In this context, our study provides a new insight: the co-expression of high levels of cytokeratin and CXCL12 is associated with better prognosis in breast cancer patients. Importantly, this observation is found in an automatic, data-driven manner. The intricate mechanism underlying this association warrants further investigation.

Our work also focuses on TNBC patients in particular, given the severity of the disease in this clinical subtype. In this context, we discovered a unique TME pattern characterized by the dense aggregation of B cells surrounded by CD4^+^ T cells, CD8^+^ T cells, macrophages, and stromal cells. This pattern closely resembles the well-known TLS structure,^56^ and we denote it as the TLS-like niche. Although the TLS-like niche is not associated with distinct prognosis across the entire discovery cohort (i.e., for all breast cancer subtypes), it is linked to better survival among TNBC patients. This association is validated in two external-validation sets, including a randomized clinical trial.^49^ We found that the expression of TLS-like niches was statistically higher among those with favorable outcomes (i.e., those with complete response) than less favorable ones. This difference was further increased in those patients receiving immunotherapy together with chemotherapy. These results suggest that TLS-like niches hold both prognostic and predictive values in TNBC patients. TLS, defined as the organized aggregation of immune cells in tumor sites resembling secondary lymphoid organs,^56^ is generally associated with promoted immune response and favorable clinical outcomes in a wide spectrum of cancers.^56^^,^^66^^,^^84^^,^^85^ However, its role in breast cancer is mixed due to the disease’s heterogeneity. TLS presence has been linked to more aggressive tumor characteristics in breast cancer, such as higher proliferation and HR negativity,^86^ as well as to the activation of tumor angiogenesis and invasion in animal models.^87^ On the other hand, TLS is associated with better survival in HER2+ patients and linked with increased lymphovascular invasion in HER2− patients.^88^ However, evidence in TNBC is more consistent, with most studies suggesting that TLS is associated with promoted immune response and better outcomes.^89^^,^^90^^,^^91^^,^^92^ Our findings align with these results but arise from machine-learning methods without explicit biological assumptions.

### Limitations of the study

Naturally, this work has some limitations. The experiments and analyses presented in this work are limited to breast cancer, although the methodology is in principle applicable to other cancers. Due to the high cost of multiplexed technology, the sample sizes in this study are small, preventing us from making stronger statistical claims. Future work should focus on applying BiGraph to larger cohorts and other disease types. On the methodological side, the Soft-WL subtree kernel proposed in this work captures small local structures (within approximately 30 μm) in the TME but shares the over-smoothing limitation common to many other graph-based algorithms. Moreover, although the Soft-WL subtree kernel provides an effective heuristic relaxation of the classical WL subtree kernel, its broader capabilities and limitations require further theoretical investigation.

## Methods

This section introduces the details of the methodology developed in our study.

### Preliminaries

We first present some key notation and definitions concerning graphs that will be necessary to introduce our methods.

An undirected graph G=(V,E) is defined by a tuple of a set of nodes V and a set of edges E⊆{(u,v)⊆V∣u≠v}. The set of nodes and the set of edges for a graph G can be written as V(G) and E(G), respectively. For each node v∈V, the set of nodes sharing an edge with it is defined as its neighborhood, denoted as N(v). The structure of a graph can be fully characterized by its adjacency matrix, A. For graphs with unweighted and undirected edges, this matrix is binary, where $A_{i,j}=1$ if $\left(v_{i},v_{j}\right)\in E\left(G\right)$. Conversely, an adjacency matrix with continuous scalar entries represents a weighted graph, where $A_{i,j}$ indicates the weight of the edge connecting nodes $v_{i}$ and $v_{j}$. Two graphs, $G_{1}$ and $G_{2}$, are deemed isomorphic if there exist a bijection $\phi :V\left(G_{1}\right)\to V\left(G_{2}\right)$, such that $\left(\phi \left(v_{i}\right),\phi \left(v_{j}\right)\right)\in E\left(G_{2}\right)$ for any $\left(v_{i},v_{j}\right)\in E\left(G_{1}\right)$.

**Figure 18:**
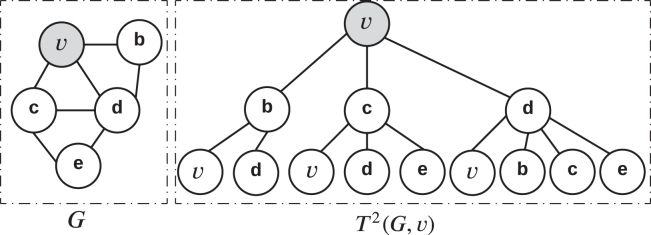
An illustration of a subtree rooted at node v (shaded) with a depth of 2

For a graph G, a subtree can be derived from a root node v∈E(G), with the neighbors N(v) serving as its children. This subtree can be expanded iteratively by adding the neighbors of the resulting nodes, increasing the depth of a subtree. A subtree rooted at v with a depth of h is denoted $T^{\left(h\right)}\left(G,v\right)$ (see Figure 18). Two subtrees are considered isomorphic if they exhibit identical node sets at every level.

### Construction of cellular graphs from TMA slides

The construction of cellular graphs involves finding a graph representation of the TME based on the spatial coordinates of the cell and their phenotypes. Each node in the cellular graph represents a single cell. The cell phenotype provides the label of each node in the graph, determined by clustering multi-channel protein expression data. In this study, we use the phenotype results directly available from the discovery^16^ and validation sets.^17^ Edges in the cellular graph represent inter-cellular connections. Previous approaches commonly employ a fixed distance threshold $\left(d_{0}\right)$ to create a binary cellular graph, where two cells are connected only if the distance between them is smaller than $d_{0}$. However, there is no consensus on the appropriate value for $d_{0}$, and various choices have been employed, such as 4,^32^ 8,^16^ and 32 μm.^15^ Additionally, there exist differences in how these distances are computed, with some works measuring distances between cell centroids and others considering minimal separation distances.

To provide a more comprehensive representation of inter-cellular interaction, our approach constructs a complete cellular graph where all pairs of cells are connected by a weighted edge, with the weight decreasing with increasing inter-cellular distance. The edge weight $w_{i,j}$ between nodes $v_{i}$ and $v_{j}$ is calculated via a Gaussian kernel,

(Equation 1) $$w_{i,j}=\exp\left({-\alpha \cdot d\left(v_{i},v_{j}\right)}^{2}\right)\text{,}$$

where $d\left(v_{i},v_{j}\right)$ represents the spatial (Euclidean) distance between centers of cells $v_{i}$ and $v_{j}$ in micrometers (μm), and α is a parameter (set as α=0.01 in our work) that determines the decay rate of the edge weights. With this choice, cells within a distance smaller than 3 μm have strong connections with edge weights higher than 0.9, while cells further than 22 μm apart have weak connections (edge weights smaller than 0.01).

In cases where a patient has multiple TMA cores, a separate cellular graph is constructed based on each core image, and these are then combined as disconnected components in a larger graph representing the entire TME for that patient. This approach easily allows for patients with multiple images while preserving the individual characteristics of each cellular graph and maintaining the entire methodology unaltered.

### Soft-WL subtree kernel

**Figure 19:**
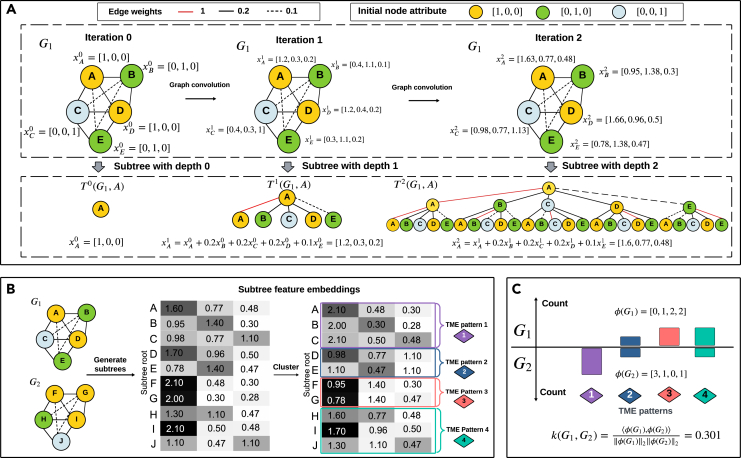
A toy example of the Soft-WL subtree kernel applied to two small cellular graphs The process of generating subtrees from the small cellular graph $G_{1}$ with five nodes. Nodes are initially assigned attributes representing the cell type (denoted by the node color), and these are iteratively updated through a graph convolution process. At each iteration, the updated node attribute serves as the feature embedding of a subtree rooted at that node. Subtrees rooted at node A with depths 0, 1, and 2 are displayed (A). Clustering of the 10 subtrees generated from two cellular graphs, $G_{1}$ and $G_{2}$, each rooted at a single node with a depth of 2. The resulting four clusters correspond to four TME patterns (B). The similarity score between $G_{1}$ and $G_{2}$, denoted as $\kappa \left(G_{1},G_{2}\right)$, is determined by the normalized inner product of their TME histograms (C).

In this section, we delve into the Soft-WL subtree kernel employed in our work, given as a relaxation of the well-known WL kernel.^45^ The WL subtree kernel, as detailed later in the section on comparative methods, relies on isomorphic subtrees as a similarity measure across graphs. The Soft-WL subtree kernel is designed to handle weighted and complete cellular graphs while providing a smoother comparison between subtrees. Figure 18 depicts a toy example of the Soft-WL subtree kernel, comprising three main steps: (1) generation of subtrees (see Figure 19A); (2) identification of TME patterns by clustering subtrees (see Figure 19B); and )3) measurement of inter-patient similarity based on the abundance of subtrees (see Figure 19C). We detail each of them below.

#### Generation of subtrees

Given that the cellular graphs are weighted and complete, the resulting subtrees are large and weighted, leading to a rare occurrence of isomorphic subtrees. To alleviate this, we compute a feature embedding for each subtree through a graph convolution process. We first add self-loops to each node, making the diagonal entries of the adjacency matrix, A, being all 1. Then, each node, $v_{i}\in V\left(G\right)$, is initially assigned an attribute given by the one-hot encoding of its cell phenotype, denoted as $x_{i}^{\left(0\right)}\in {\left\{0,1\right\}}^{m}$, where m is the number of unique cell phenotypes. The node attribute $x_{i}^{\left(h\right)}\in R^{m}$ is then iteratively updated at each $h^{th}$ iteration as follows:

(Equation 2) $$x_{i}^{\left(h\right)}=x_{i}^{\left(h-1\right)}+\sum_{v_{j}\in V\setminus \left\{v_{i}\right\}}x_{j}^{\left(h-1\right)}A_{i,j}\text{.}$$

Intuitively, this graph convolution process updates the feature of node $v_{i}$ to incorporate not only its phenotype information but also that of its neighbors. Thus, $x_{i}^{\left(h\right)}$ provides the feature embedding of the subtree rooted at $v_{i}$ with a depth of h, and it encodes the structure of a local cellular neighborhood around cell $v_{i}$.

Note that the cellular neighborhood does not have a clear boundary, but one can decide on a boundary by applying a threshold τ to the maximum considered distance (or, equivalently, to the minimal weight). More specifically, Equation 2 can be rewritten as

(Equation 3) $$x_{i}^{\left(h\right)}=x_{i}^{\left(0\right)}{\left(A^{h}\right)}_{i,i}+\sum_{v_{j}\in V\setminus \left\{v_{i}\right\}}x_{j}^{\left(0\right)}{\left(A^{h}\right)}_{i,j}\text{,}$$

where $A^{h}$ denotes the $h^{\text{th}}$ power of the adjacency matrix A, and ${\left(A^{h}\right)}_{i,j}$ denotes the influence of a node $v_{j}$ to $x_{i}^{\left(h\right)}$. Then, any node $v_{j}$ with ${\left(A^{h}\right)}_{i,j}>\tau$ is considered part of the cellular neighborhood. This hyperparameter, τ, is user defined, and we set it as 0.01 in our experiments. In this way, N subtrees can be generated from a cellular graph with N cells, each rooted at a single cell. In turn, each subtree is associated with a feature embedding derived from the graph convolution process above, representing the structure of the cellular neighborhood around each cell.

#### Identification of TME patterns

Unlike the traditional WL kernel,^45^ which focuses on the distribution of subtrees as a similarity measure between graphs, the Soft-WL subtree kernel clusters similar subtrees $T^{\left(h\right)}\left(G,v_{i}\right)$ based on their feature embeddings $x_{i}^{\left(h\right)}$. Moreover, instead of comparing subtrees embedding themselves, we will compare the distribution of recurring cell patterns across patients (or graphs) as characterized by their embeddings.

To this end, we employ a clustering algorithm across subtrees extracted from the entire discovery set. Although the clustering method can be general, we employ the PhenoGraph algorithm^47^ with hyperparameter k=100. This algorithm is not sensitive to the choice of k in the context of single-cell data. Each resulting cluster is considered a TME pattern, characterizing subtrees with similar embeddings. Furthermore, each TME pattern is associated with a signature, denoted by S and given by the cluster centroid. More precisely, for each c out of C clusters,

(Equation 4) $$S\left(c\right)=\frac{1}{\left|\left\{T^{\left(h\right)}\left(G,v_{i}\right)\in T|l\left(v_{i}\right)=c\right\}\right|}\sum_{i:l\left(v_{i}\right)=c}x_{i}^{\left(h\right)}\text{,}$$

where $x_{i}^{\left(h\right)}$ is the subtree feature embedding of node (cell) $v_{i}$, T denotes the set of subtrees derived from the entire discovery set, and l:T→{1,…,C} denotes a function mapping a subtree to its assigned cluster identity.

#### Inter-patient similarity

The Soft-WL subtree kernel calculates the inter-patient similarity by comparing the abundance of TME patterns across patients. Let $\phi \left(G_{i}\right)$ be the histogram of TME patterns from each subject, represented by its graph $G_{i}$. The similarity between graphs $G_{i}$ and $G_{j}$ is given by

(Equation 5) $$\kappa \left(G_{i},G_{j}\right)=\frac{\left\langle \phi \left(G_{i}\right),\phi \left(G_{j}\right)\right\rangle }{\left\|\phi \left(G_{i}\right){\|}_{2}\right\|\phi \left(G_{j}\right){\|}_{2}}\text{,}$$

where ${\left\|\cdot \right\|}_{2}$ denotes the $l_{2}$ (Euclidean) norm. The normalization ensures that the similarity score for any pairs of patients $\kappa \left(G_{i},G_{j}\right)\in \left[0,1\right]$, with identical graphs achieving a maximal similarity of 1. The hyperparameter h, implicit in the definition of $\phi \left(G_{i}\right)$, controls the depth of subtrees and determines the size of the cellular neighborhoods. We set it as h=2 in the experiments. The resulting cellular neighborhoods contain a median of 16 cells, with a median radius (defined by the maximum distance between a subtree root and the cells within that cellular neighborhood) of 30 μm.

### Population graph and community detection

In the previous section, patient-specific graphs were constructed to characterize the spatial distributions of cells in their TMEs. We now move on to expanding on the population-level graph.

The population graph is constructed to encode the similarities *across* patients. Each node of the population graph represents a patient, and every patient is connected to potentially all other patients through edges with scalar weights given by the Soft-WL subtree kernel. Thus, the population graph can be represented by the kernel matrix $K\in R^{N\times N}$ for the set of N patients $\left\{G_{1},G_{2},\ldots ,G_{N}\right\}$ in the discovery set, and each entry $K_{ij}$ is given by $K_{ij}=\kappa \left(G_{i},G_{j}\right)$ (see Equation 5).

Before proceeding to perform community detection, we preprocess the population graph as per the instructions in Levine et al.^47^ by (1) identifying the $k^{⋆}$-nearest neighbors ($k^{⋆}$-NN) for each node and (2) replacing the edge weight between two patients by the IoU of their respective $k^{⋆}$-NN sets. The Louvain community detection method^48^ (Python package “networkx”^93^) is then employed to detect communities within the population graph, where each community represents a patient subgroup characterized by high intra-group similarities in their TMEs. We set the hyperparameter $k^{⋆}=30$ throughout our experiments. Although this hyperparameter impacts the granularity of the resulting communities, the patient grouping remains quite consistent across various choices of $k^{⋆}$ (see Figure S2).

### Identification of characteristic patterns

The pattern histogram is normalized on a per-patient basis by dividing the occurrence of each pattern by the sum of occurrences of all patterns within that patient, yielding the proportion of each TME pattern in individual patients (see Figure S3). Consequently, the Hodges-Lehmann statistic^55^ is employed to compare the proportions of specific TME patterns inside and outside a patient subgroup (Figure S4). A higher Hodges-Lehmann statistic indicates the increased expression of the studied pattern in a patient subgroup. A TME pattern is deemed characteristic if its Hodges-Lehmann statistic surpasses 50% of the maximum value within that patient subgroup, as highlighted in colored boxplots in Figure S4.

### Prognosis analysis

The K-M estimator^51^ is used to generate survival plots for each patient subgroup. For the discovery and inner-validation set, disease-specific survivals are considered. On the other hand, overall survival is considered for the external-validation set-1, since the disease-specific survival data are not available. The multivariate log rank test^52^ is used to compare their survivals. A Cox proportional hazard regression model^53^ is used to calculate hazard ratios for each patient subgroup, with patients outside each subgroup serving as the baseline. In assessing the prognostic impact of a specific TME pattern, patients are stratified into positive and negative groups based on the expression of the target TME pattern, where a proportion of 1% is used as the threshold for categorization. The survival outcomes of positive and negative patients are compared using the pairwise log rank test. All these prognosis-related statistical methods are conducted with the Python implementation from “lifelines.”^94^

### Validation and generalization

The robustness and generalization of the major findings in the discovery set are rigorously assessed in both an inner-validation set and two external-validation sets. The validation process encompasses mapping at the cellular, subtree, and patient levels. We detail each of them in the following.

#### Cell phenotyping mapping

Cell phenotype mapping is only needed for the external-validation sets since they employ different cell phenotyping systems from that in the discovery set. Moreover, the external-validation sets also employ different antigen marker panels, with approximately 50% of the antigens shared with the discovery set. Each cell, for all datasets, is characterized by a continuous feature vector representing the expression profiling of the *shared* antigens. For a cell from either of the external-validation sets, mapping is achieved by assigning it to the phenotypically closest cell phenotype (i.e., a cluster of cells) defined within the discovery set. The phenotypic distance between a cell and a cell cluster is given by the Euclidean distance between the cell’s feature vector and the cluster centroid.

#### TME pattern mapping

For each new patient in the validation sets with the corresponding cellular graph, subtree generation and feature computation are carried out analogously to that described in Methods. Instead of re-conducting clustering on the subtrees from the validation sets to find new patterns, they are mapped to the closest TME pattern previously defined within the discovery set (i.e., allowing us to assign a new patient to a predefined subgroup). The distance between a subtree and a TME pattern is given by the Euclidean distance between the feature vectors of the subtree and the pattern’s signature (or cluster centroid).

#### Patient subgroup mapping

Once TME patterns are mapped to those found in the discovery set, the similarity between patients across sets can be calculated using Equation 5. Given a new patient from the validation sets, its top three most similar patients in the discovery set are identified. The new patient is then mapped to the patient subgroup via majority voting (weighted by inter-patient similarity) among the three nearest neighbors. In this way, a corresponding patient subgroup, denoted as ${\text{Si}}^{\prime }$, can be identified for every group Si, both in the inner- and external-validation sets. Survival analysis is then performed to discern whether a mapped patient subgroup mirrors the survival outcome of its phenotypically similar patient subgroup defined within the discovery set.

### Comparative methods

Finally, this section details alternative methods for measuring inter-patient similarity. The overall kernel method (as in Equation 5) will be maintained, but the representation of every patient, ϕ(G), will vary for each method.

• Abundance of cell types. In this method, ϕ(G) represents the histogram of cell phenotypes within the cellular graph of each patient.
• Pairwise proximity of cell phenotypes. Consider a dataset with n unique cell phenotypes. ϕ(G) is a feature vector with $\frac{n\left(n-1\right)}{2}$ entries, each corresponding to the average proximity score between a pair of cell phenotypes. Formally, consider the phenotype of any given cell $v_{i}$ to be $p\left(v_{i}\right)\in \left\{1,\ldots ,m\right\}$, the proximity between phenotypes a and b is given by

(Equation 6) $$\text{proximity}\left(a,b\right)=\frac{1}{N_{a,b}}\sum_{\begin{array}{l} \left(v_{i},v_{j}\right)\in E\left(G\right)\\ p\left(v_{i}\right)=a,p\left(v_{j}\right)=b \end{array}}\exp\left({-\alpha \cdot d\left(v_{i},v_{j}\right)}^{2}\right)\text{,}$$

where $N_{a,b}=\left|\left\{\left(v_{i},v_{j}\right)\in E\left(G\right):p\left(v_{i}\right)=a,p\left(v_{j}\right)=b\right\}\right|$ is the number of pairs of cells of phenotypes a and b, and $d\left(v_{i},v_{j}\right)$ is the spatial distance (in μ m) between $v_{i}$ and jv.

• WL subtree kernel.^45^ This kernel measures the similarity between two graphs by counting the occurrences of isomorphic subtrees in them. Weighted cellular graphs are converted to binary graphs by applying a threshold of 0.01 to the edge weights. This threshold connects two cells if the distance between their centroids is less than 21 μ m. Identifying isomorphic subtrees involves a color refinement procedure, where node colors are iteratively updated based on their neighbors. Each node is initially assigned a color based on its cell phenotype, denoted as $\sigma^{0}\left(v\right)$. The node color can be taken from a discrete alphabet or can be an integer. The node color is subsequently updated as follows:

(Equation 7) $$\sigma^{h}\left(v\right)=\text{relabel}\left(\sigma^{h-1}\left(v\right),\text{sort}\left(\left\{\sigma^{h-1}\left(u\right)|u\in N\left(v\right)\right\}\right)\right)\text{,}$$

where $\sigma^{h}\left(v\right)$ is the node color at iteration h, and N(v) represents the neighbors of v. The function sort collects and sorts the multiset of colors of N(v), and the function relabel maps the pair $\left(\sigma^{h-1}\left(v\right),\text{sort}\left(\sigma^{h-1}\left(u\right)|u\in N\left(v\right)\right)\right)$ to a unique color not used in previous iterations.

Two nodes with the same color after iteration h imply the isomorphism of the two subtrees rooted at these nodes with depth h. Notably, the color refinement is conducted universally for all the graphs in the dataset rather than per patient, ensuring a consistent color system across all graphs. In this case, the feature ϕ(G) for each patient represents the histogram of unique colors in their TME, and two slightly different formulations are considered.

Let $\sigma_{h,i}$ denote the $i^{th}$ unique color appearing at the $h^{th}$ iteration of color refinement. Furthermore, consider the set of these $C_{h}$ unique colors at the $h^{th}$ iteration, $\Sigma_{h}=\left\{\sigma_{h,1},\ldots ,\sigma_{h,C_{h}}\right\}$. Then, consider $c\left(G,\sigma_{hi}\right)$ as the number of occurrences of color $\sigma_{h,i}$ in the graph G, the first formulation for the feature of G is given by

(Equation 8) $$\phi \left(G\right)=\left[c\left(G,\sigma_{0,1}\right),\ldots ,c\left(G,\sigma_{0,C_{0}}\right),\ldots ,c\left(G,\sigma_{h,1}\right),\ldots ,c\left(G,\sigma_{h,C_{h}}\right)\right]\text{.}$$

The second formulation only considers the colors from the last iteration of color refinement, given by

(Equation 9) $$\phi \left(G\right)=\left[c\left(G,\sigma_{h,1}\right),\ldots ,c\left(G,\sigma_{h,C_{h}}\right)\right]\text{.}$$

The hyperparameter h controls the depth of subtrees, similar to its counterpart in the Soft-WL subtree kernel. We set it to 2 in our experiments, consistent with the setting used for the Soft-WL subtree kernel. The WL subtree kernel is implemented with the Python package “Grakel.”^95^

## Resource availability

### Lead contact

Requests for further information and resources should be directed to and will be fulfilled by the lead contact, Jeremias Sulam (jsulam1@jhu.edu).

### Materials availability

This study did not generate new materials.

### Data and code availability

• The multiplexed image data and corresponding clinical data used in this work are publicly available at Zenodo Data: https://doi.org/10.5281/zenodo.5850952 (Danenberg et al.^16^), https://doi.org/10.5281/zenodo.3518284 (Jackson et al.^17^), and Zenodo Data: https://doi.org/10.5281/zenodo.7990870 (Wang et al.^49^).
• Our source code is available on GitHub (https://github.com/Sulam-Group/BiGraph4TME.git) and has been archived at Zenodo.^96^
• Any additional information required to reanalyze the data reported in this paper is available from the lead contact upon request.

## Acknowledgments

This study is supported in part by the 10.13039/100000002National Institutes of Health grant R01CA138264 and NSF CAREER Award CCF 2239787.

## Author contributions

Conceptualization, Z.W. and J.S.; methodology, Z.W., C.A.S.-M., A.S.P., and J.S.; investigation, Z.W., C.A.S.-M., A.S.P., and J.S.; writing – original draft, Z.W.; writing – review and editing, Z.W. C.A.S.-M., A.S.P., and J.S.; funding acquisition, J.S. and A.S.P.; supervision, J.S. and A.S.P.

## Declaration of interests

The authors declare no competing interests.

## Declaration of generative AI and AI-assisted technologies

During the preparation of this work, the author(s) used the large language model (ChatGPT 3.5) in order to improve conciseness. After using this tool or service, the author(s) reviewed and edited the content as needed and take(s) full responsibility for the content of the publication.

## Supplemental information

Document S1. Figures S1–S9

Document S2. Article plus supplemental information
