# Supplementary material for: Bi-level graph learning unveils prognosis-relevant tumor microenvironment patterns in breast multiplexed digital pathology
Source: Patterns (N Y). 2025 Feb 11;6(3):101178. doi: 10.1016/j.patter.2025.101178 (PMC11962943; doi:10.1016/j.patter.2025.101178)
Supplement: Document S1. Figures S1–S9 [file mmc1.pdf]

Patterns, Volume *6*

Supplemental information

Bi-level graph learning unveils prognosis-relevant tumor microenvironment patterns in breast multiplexed digital pathology

Zhenzhen Wang, Cesar A. Santa-Maria, Aleksander S. Popel, and Jeremias Sulam

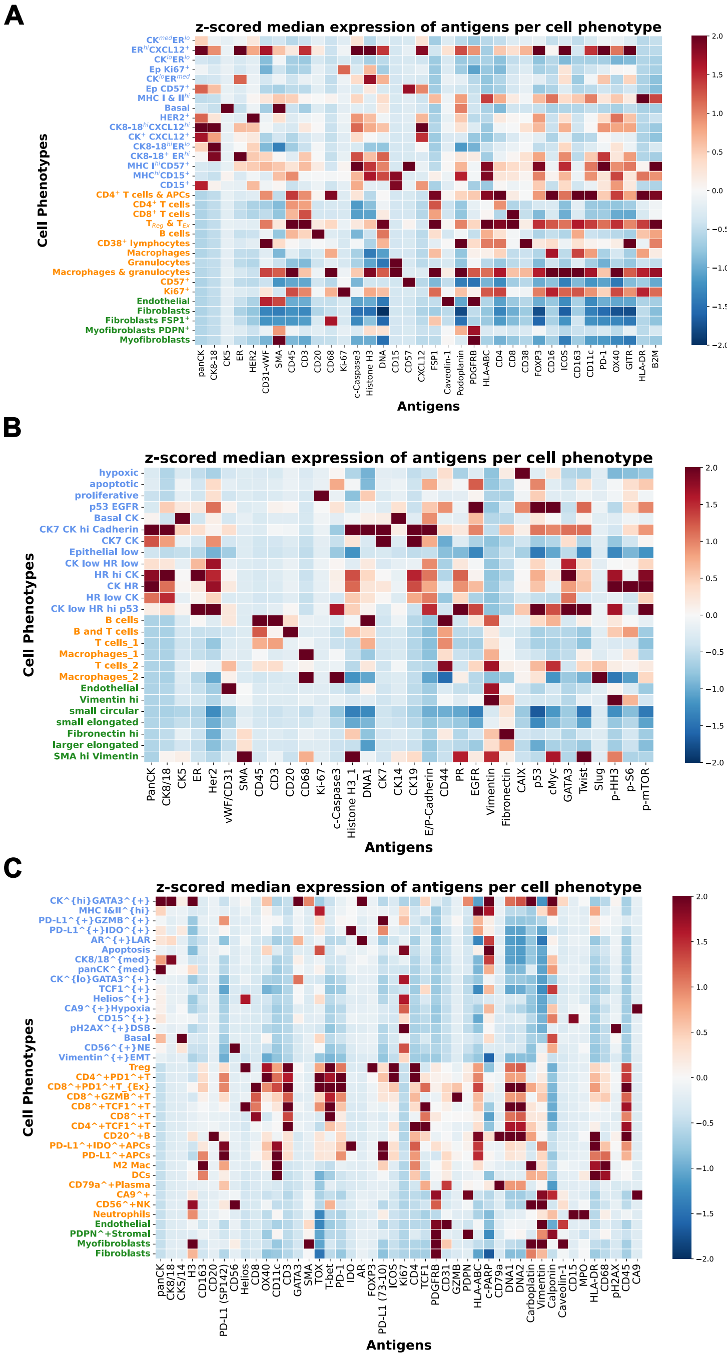

Figure S1. Cell phenotyping system of the datasets used in this study. The median antigen profiling of different cell phenotypes in the discovery set (A), external validation set-1 (B), and external validation set-2 (C). The y-axis colors indicate the cell categories (blue: tumor cells, orange: immune cells, green: stromal cells).

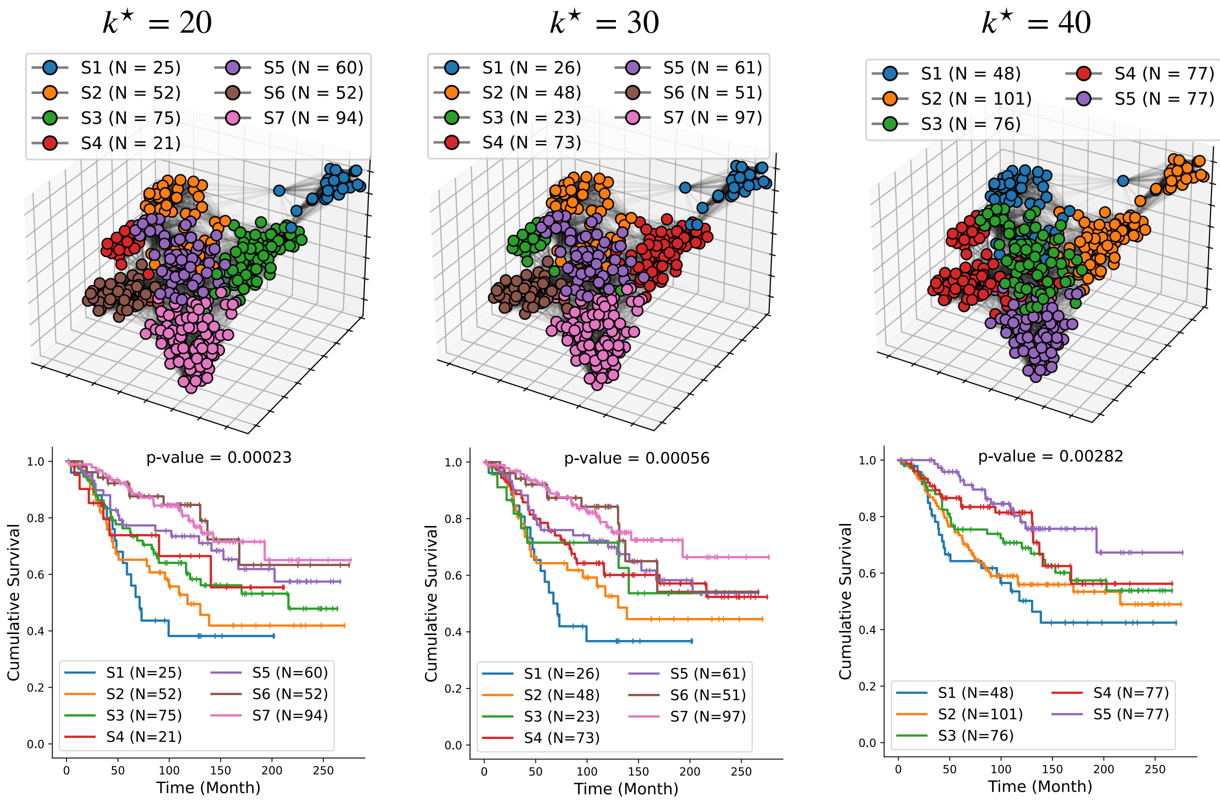

Figure S2. Clustered patient subgroups with different choices of hyperparameter $k^\star$. The first row displays population graphs with nodes colored by patient subgroup IDs, and the second row shows the survival plots of patient subgroups. Multi-variate log-rank test is used to compare their survivals, and the p-values are shown on the titles.

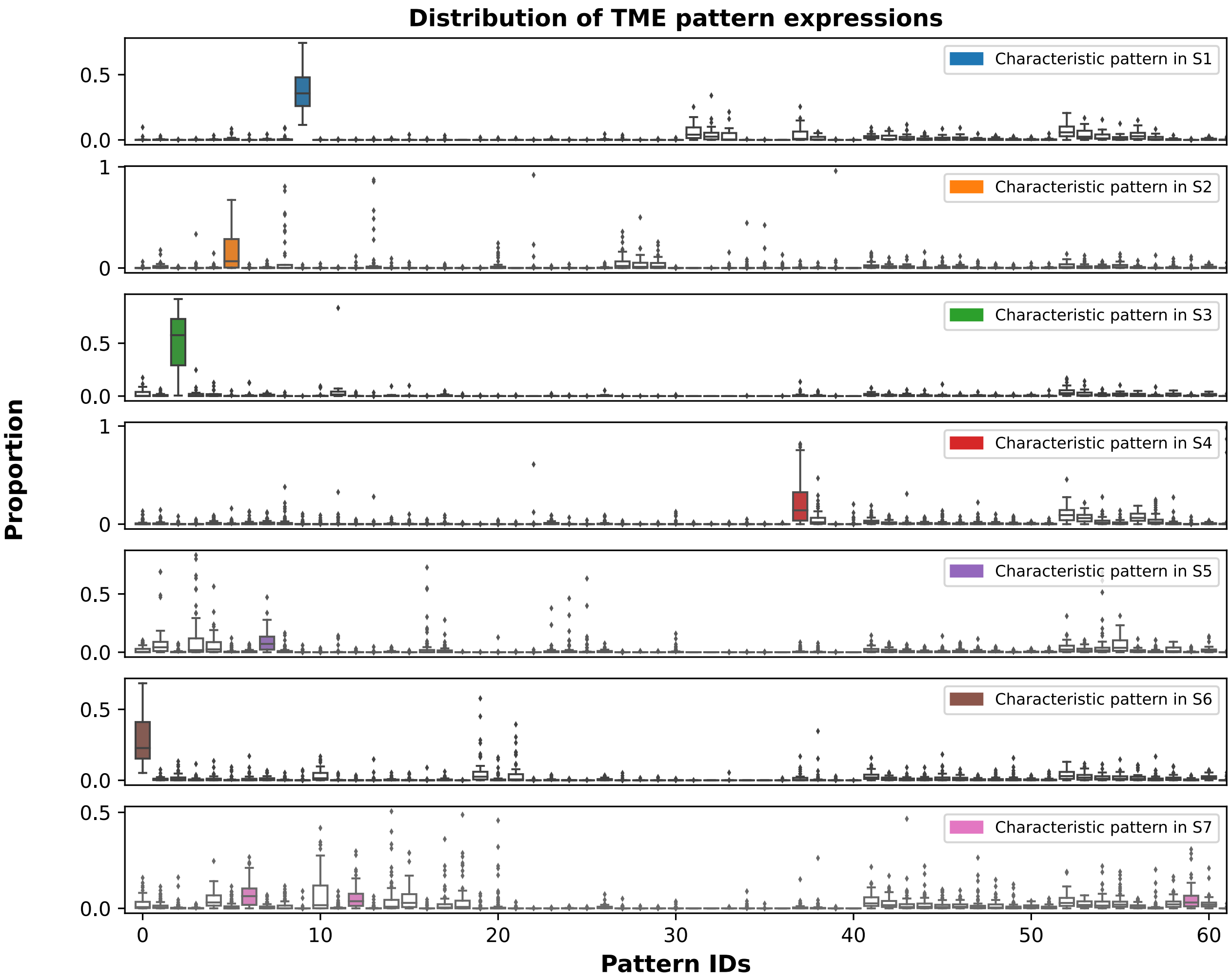

Figure S3. Distribution of TME pattern expressions in each of the seven patient subgroups in the discovery set. The proportion of each TME pattern within an individual patient is calculated by normalizing the corresponding TME pattern histogram. The distribution of these proportions across patient subgroups is represented using box plots. Characteristic patterns are highlighted by colored box plots.

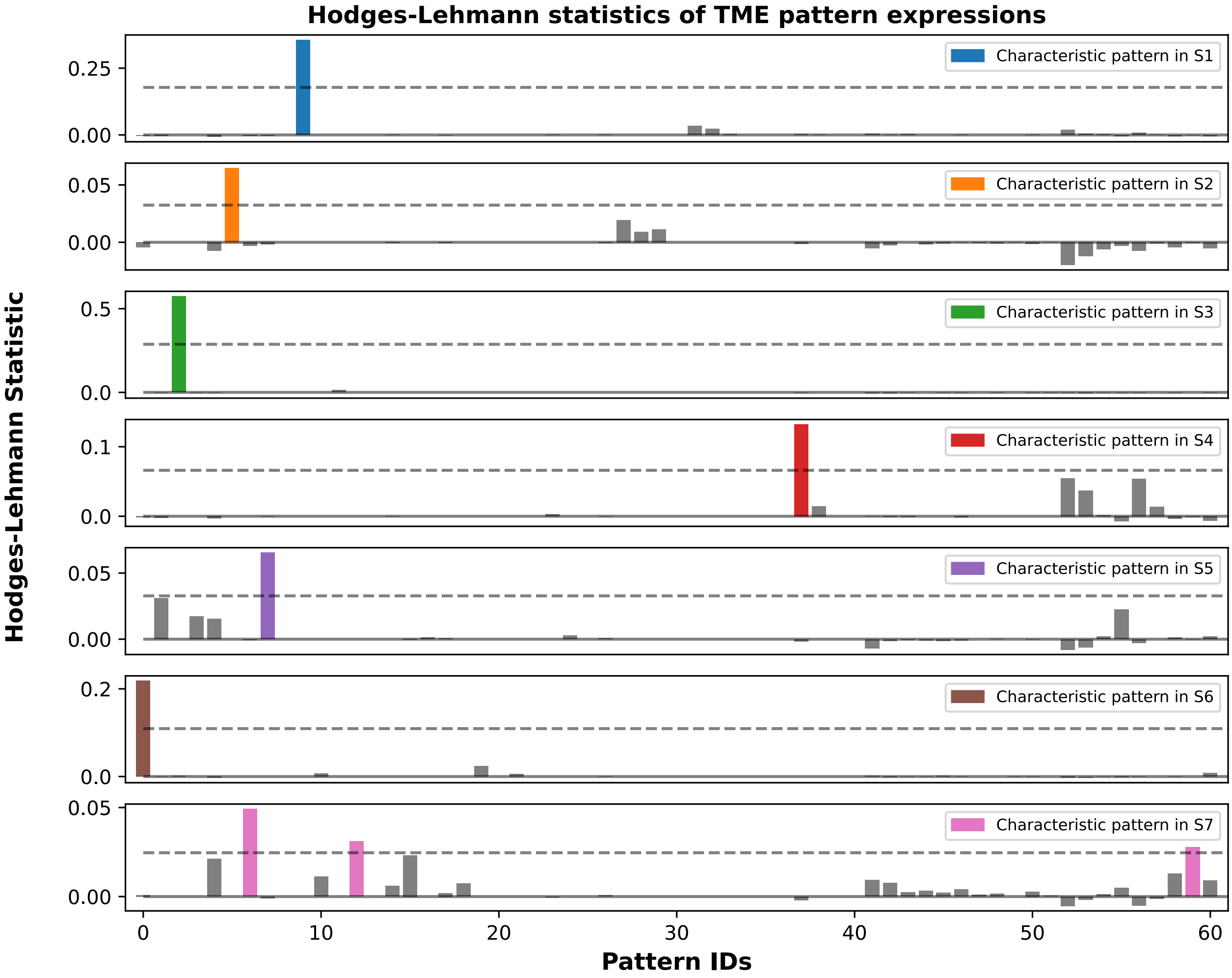

Figure S4. Hodge-lehmann statistics of TME pattern expressions in each of the seven patient subgroups. For each TME pattern, the distribution of its proportion among patients inside and outside that subgroup is compared using the Hodges–Lehmann statistic. A larger Hodges–Lehmann statistic implies an over-expression of that particular pattern in that patient subgroup. A TME pattern is deemed “characteristic” in a patient subgroup if the Hodges–Lehmann statistic exceeds 50% of the maximized value within that patient subgroup, highlighted by colored bar plots.

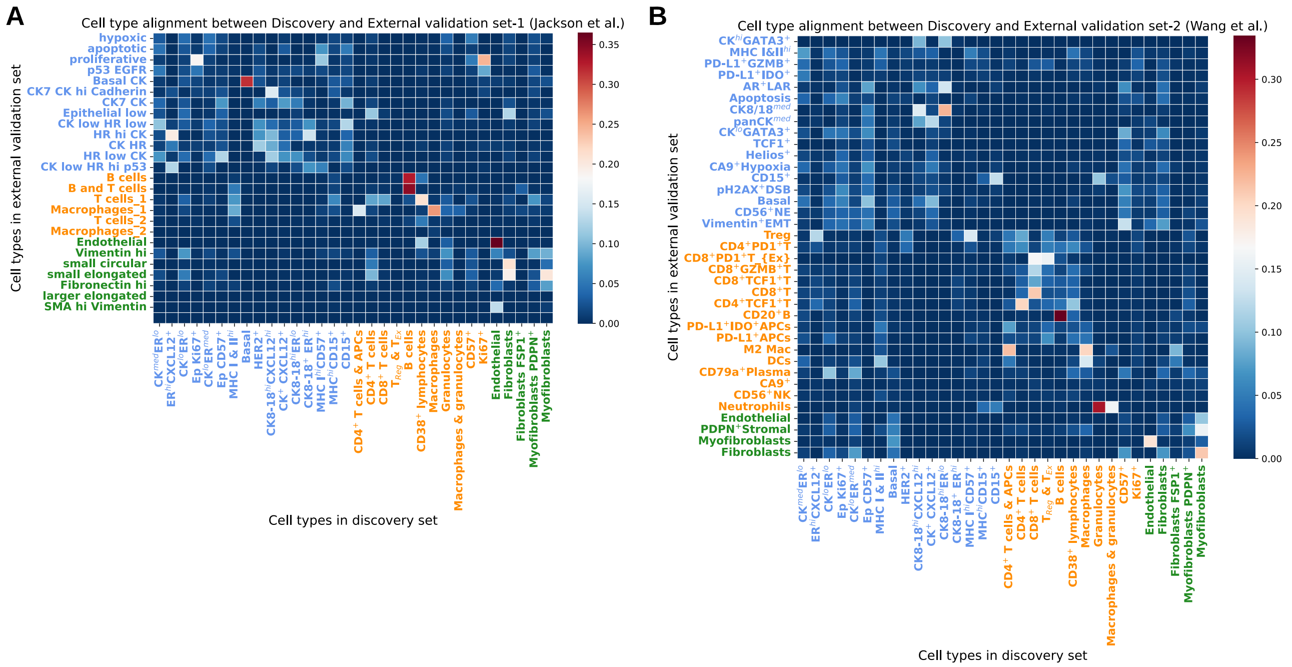

Figure S5. Alignment of cell phenotyping systems between discovery and external validation sets. Each cell within the external validation set is associated with two distinct phenotypes: the original label, as designated in the external phenotyping system, and the label assigned within the phenotyping system of the discovery set, determined through cell phenotype mapping. The Intersection over Union (IoU) is computed for each pair of cell phenotypes derived from these two distinct phenotyping systems, and the results are visually represented in the heatmap. The alignment between the discovery set and external validation set-1 (A). The alignment between the discovery set and external validation set-2 (B).

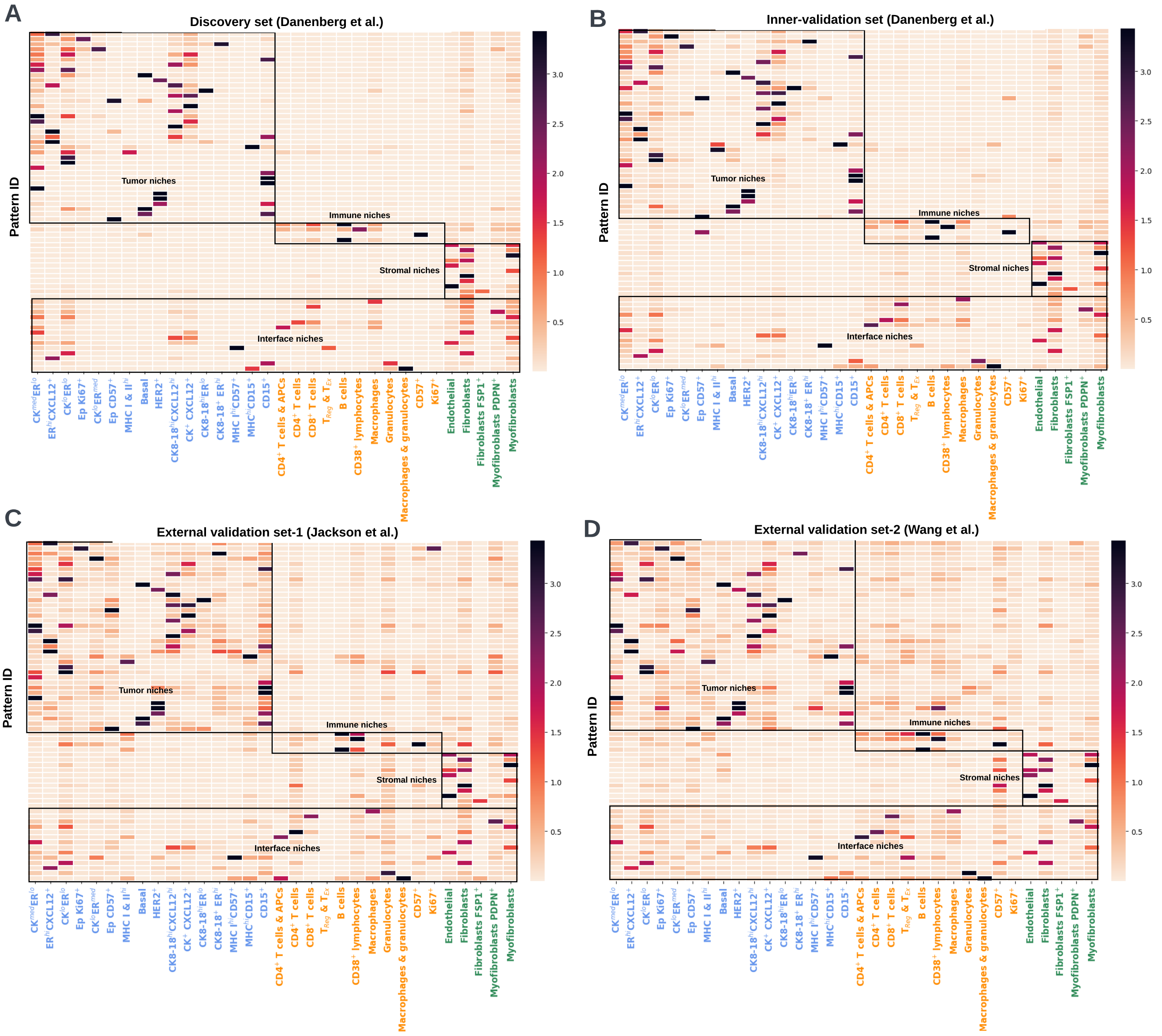

Figure S6. ignature maps of 66 TME patterns across different datasets. The signature map (cluster centroids) of 66 TME patterns identified in the discovery set (A). In the validation sets, each subtree is mapped to one of these 66 predefined TME patterns. The figures show the signature maps (cluster centroids) of the 66 TME patterns in the inner-validation set (B), external validation set-1 (C), and external validation set-2 (D)

.

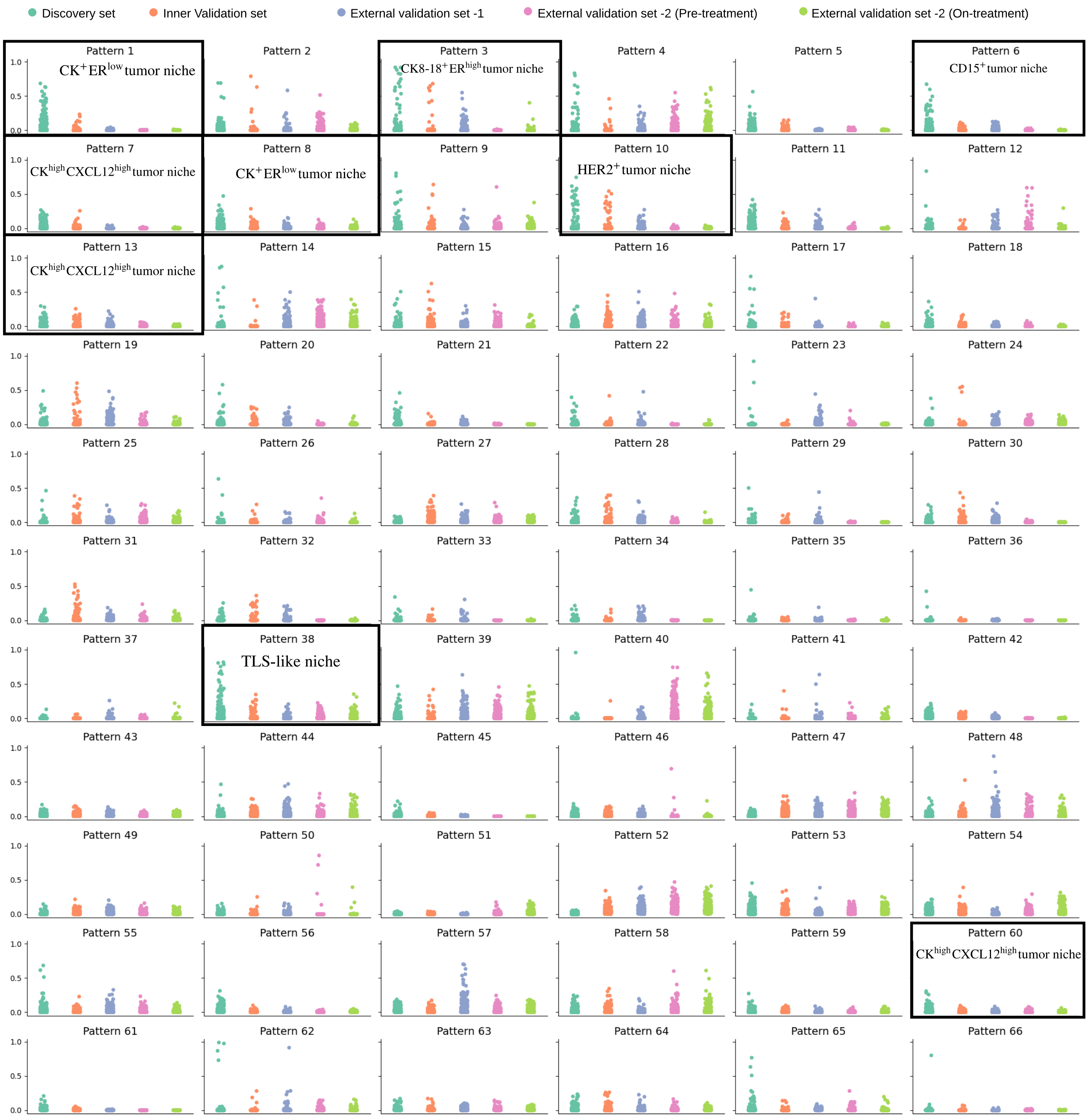

Figure S7. Distribution of 66 TME patterns across different datasets. Each panel presents the distribution of a specific TME pattern. The x-axis represents the datasets, while the y-axis shows the proportion of the TME pattern. Each small circle corresponds to the proportion of a specific TME pattern in an individual patient. Different colors represent corresponding datasets: cyan for the discovery set, orange for the inner validation set, blue for external validation set-1, purple-red for external validation set-2 (pre-treatment samples), and lime for external validation set-2 (on-treatment samples).

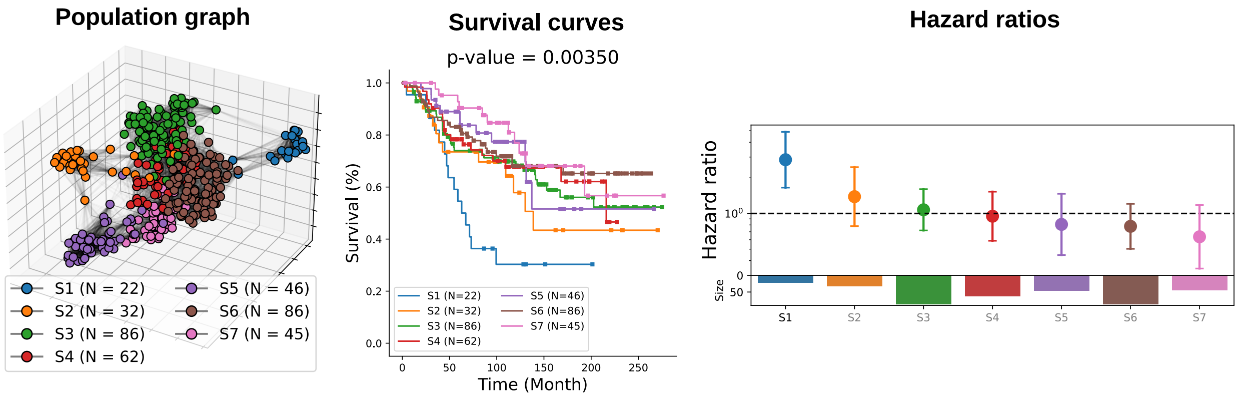

Figure S8. Risk stratification results from WL subtree kernel (accumulating similarities from all previous iterations). From left to right, the figure shows a population graph with nodes colored by patient subgroup labels, K-M survival plots of patient subgroups, and hazard ratios estimated by the Cox proportional model.

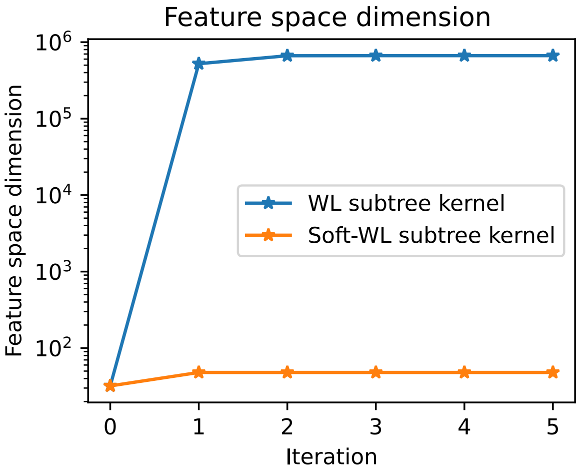

Figure S9. Feature space dimension of WL and Soft-WL subtree kernels. For the WL subtree kernel, the feature space dimension refers to the number of unique colors of each iteration of color refinement. For the soft-WL subtree kernel, the feature space dimension refers to the number of TME patterns at each iteration of graph convolution.
